# Expanding the Application of Sentinel-2 Chlorophyll Monitoring across United States Lakes

**DOI:** 10.3390/rs16111977

**Published:** 2024-05-30

**Authors:** Wilson B. Salls, Blake A. Schaeffer, Nima Pahlevan, Megan M. Coffer, Bridget N. Seegers, P. Jeremy Werdell, Hannah Ferriby, Richard P. Stumpf, Caren E. Binding, Darryl J. Keith

**Affiliations:** 1 U.S. Environmental Protection Agency Office of Research and Development, Research Triangle Park, NC 27711, USA;; 2 NASA Goddard Space Flight Center, Ocean Ecology Lab, Greenbelt, MD 20771, USA; 3 Science Systems and Applications, Inc., Lanham, MD 20706, USA; 4 National Oceanic and Atmospheric Administration, NESDIS Center for Satellite Applications and Research, College Park, MD 20740, USA; 5 Global Science & Technology, Inc., Greenbelt, MD 20770, USA; 6 Morgan State University, Baltimore, MD 21251, USA; 7 Tetra Tech, Research Triangle Park, NC 27709, USA; 8 National Oceanic and Atmospheric Administration, National Centers for Coastal Ocean Science, Silver Spring, MD 20910, USA;; 9 Environment and Climate Change Canada, Water Science and Technology Directorate, Burlington, ON L7S 1A1, Canada;; 10 U.S. Environmental Protection Agency Office of Research and Development, Narragansett, RI 02882, USA

**Keywords:** chlorophyll *a*, eutrophication, Sentinel-2, remote sensing, water quality monitoring, lakes

## Abstract

Eutrophication of inland lakes poses various societal and ecological threats, making water quality monitoring crucial. Satellites provide a comprehensive and cost-effective supplement to traditional in situ sampling. The Sentinel-2 MultiSpectral Instrument (S2 MSI) offers unique spectral bands positioned to quantify chlorophyll *a*, a water-quality and trophic-state indicator, along with fine spatial resolution, enabling the monitoring of small waterbodies. In this study, two algorithms—the Maximum Chlorophyll Index (MCI) and the Normalized Difference Chlorophyll Index (NDCI)—were applied to S2 MSI data. They were calibrated and validated using in situ chlorophyll *a* measurements for 103 lakes across the contiguous U.S. Both algorithms were tested using top-of-atmosphere reflectances (*ρ*_t_), Rayleigh-corrected reflectances (*ρ*_s_), and remote sensing reflectances (*R*_*rs*_). MCI slightly outperformed NDCI across all reflectance products. MCI using *ρ*_t_ showed the best overall performance, with a mean absolute error factor of 2.08 and a mean bias factor of 1.15. Conversion of derived chlorophyll *a* to trophic state improved the potential for management applications, with 82% accuracy using a binary classification. We report algorithm-to-chlorophyll-*a* conversions that show potential for application across the U.S., demonstrating that S2 can serve as a monitoring tool for inland lakes across broad spatial scales.

## 1. Introduction

Eutrophication has emerged as a major ecological threat to coastal and inland lakes across the United States (U.S.) and the world [1]. It occurs when nutrients from agriculture and urban development create conditions favorable for phytoplankton proliferation [2]. These blooms can limit availability of light and nutrients to aquatic plants and cause hypoxia, resulting in death of fish and other aquatic animals [3]. Phytoplankton blooms are often dominated by cyanobacteria, which have the potential to produce toxins [4], presenting risks to aquatic recreation, municipal drinking water security, and wildlife and livestock safety [5]. Chlorophyll *a* (chl *a*), a photosynthetic pigment, serves as an indicator for both algae and cyanobacteria; chl *a* concentration (expressed here in μg L^−1^) is often used to assess eutrophication and trophic state in aquatic systems [6]. Additionally, reducing chl *a* has been proposed as a method to limit disinfection byproducts in finished drinking water, compounds with potential human health implications that form when source waters high in organic matter are halogenated [7].

Given the multitude of ecological [8] and societal [9] impacts stemming from eutrophication, it is crucial for managers to know when, where, and to what degree eutrophic conditions exist. This information is often provided in the form of trophic state, a categorical system of nutrient loading and resulting phytoplankton abundance that can be defined by chl *a* concentration [6]. Chl *a* monitoring is performed to meet regulatory requirements [7]—including for total maximum daily loads (TMDLs) [10]—and may inform short-term responses and long-term management strategies to mitigate eutrophication. This monitoring is typically achieved through in situ measurements. Financial costs limit the frequency and spatial coverage of in situ sampling, often inhibiting a holistic determination of eutrophication and assessment of intra-lake heterogeneity in trophic status [11,12]. Satellite remote sensing has been introduced as a strategy to address such constraints to water quality monitoring [13]. Satellite-derived chl *a* can provide more geographically and temporally dense data when used to complement in situ measurements. However, there remains the need to choose a well-validated chl *a* satellite algorithm that could become a standardized product and ease the use of using satellite-derived chl *a* for water quality monitoring, especially for small lakes.

At high concentrations, chl *a* displays a distinct reflectance peak in the red edge spectral range near 700 nm, the height of which is proportional to the concentration of chl *a* [14]. The primary satellite sensors used for inland chl *a* detection historically have been moderate-resolution sensors such as the European Space Agency (ESA) Envisat MEdium Resolution Imaging Spectrometer (MERIS) and the Sentinel-3 (S3) Ocean and Land Colour Instrument (OLCI), both of which offer spatial resolutions of 300 m. The National Aeronautics and Space Administration (NASA) Moderate Resolution Imaging Spectroradiometer (MODIS), which offers 250- to 1000-m resolution depending on the spectral bands selected, has also been used. These platforms generally deliver data with a temporal frequency of 1 to 3 days and have the spectral bands necessary for detecting chl *a*; specifically, in addition to blue and green bands traditionally utilized for open ocean chl *a* retrievals, they also offer red and near-infrared bands, which are often considered more suitable for optically complex waters and high chl *a* concentration ranges. While MERIS [15–17], OLCI [18], and MODIS [19,20] have been successfully applied to large inland lakes (>1 km wide) for water quality monitoring, their relatively coarse spatial resolution precludes their use in smaller (≤1 km wide) lakes. Additionally, even in larger lakes, viewing of near-shore waters is limited since shore-adjacent pixels have a high likelihood of contamination from stray light [21]. Incidentally, the near-shore environment is where cyanobacteria biomass tends to accumulate—particularly in windy conditions [22]—and where most human recreational swimming exposure occurs [23]. For these reasons, efforts have been made to utilize finer-scale sensors designed for terrestrial science and applications for aquatic chl *a* detection. These sensors include the jointly managed NASA and U.S. Geological Survey (USGS) Landsat series, which has a spatial resolution of 30 m [24–29], and in some cases even finer-scale commercial platforms such as Maxar’s WorldView-2 and WorldView-3 with 1-to-2 m resolution [30,31] and Planet’s RapidEye with 5 m resolution [32,33].

While finer-scale sensors provide some ability to identify chl *a* presence at a higher spatial resolution, they often do not possess the spectral bands required to quantify the reflectance peak of chl *a* in the red edge region. In addition, there are complications related to atmospheric correction and the portability of empirical associations with band ratio algorithms [34]. Thus, while chl *a* algorithms developed for such sensors may perform well when tuned for individual lakes, efforts to scale them to broader applications across ecosystems are challenging and limited. ESA’s MultiSpectral Instrument (MSI) sensors aboard the Sentinel-2A and −2B (S2) satellites offer an opportunity to overcome these limitations, including three bands in the red edge region. Since their launch in 2015 and 2017, respectively, these sensors have provided 10-to-60 m resolution, depending on the spectral band, with a combined 5-day revisit frequency. Previous studies indicate S2 may be well suited for deriving chl *a* in lakes and reservoirs, for example in Europe [35–37], South America [38–40], Asia [41–44], the U.S. [45,46], and across a global set of lakes, with a rigorous validation across North America [47,48]. However, these studies tend to focus on individual or a limited number of lakes. With the exception of [48], there is a paucity of work scaling S2 algorithms to multiple inland waters at broad scales.

A standard remote sensing method to detect chl *a* in lakes across the contiguous U.S. (CONUS) would reduce the need to develop and validate individual algorithms for specific lakes. Here, we test the application of two chlorophyll algorithms—the Maximum Chlorophyll Index (MCI) [49] and the Normalized Difference Chlorophyll Index (NDCI) [50]—using S2 MSI data, and assess their performances across 103 U.S. lakes and reservoirs. The MCI and NDCI were selected since both are adaptable to S2 and widely used. Baseline subtraction methods such as the MCI are often considered favorable due to their effective handling of sun glint [34]. The MCI is unique among such algorithms since it is retrievable using S2 bands, unlike other well-known baseline subtraction algorithms such as Fluorescence Line Height [15], Cyanobacteria Index [51], and Maximum Peak Height [52]; these algorithms require additional spectral bands not offered by S2. Additionally, the MCI is available as a built-in tool within the Sentinel Application Platform (SNAP: https://step.esa.int/main/download/snap-download [accessed 1 May 2024])—free software publicly available from ESA—making it relatively easy to implement, and accessible to stakeholders with limited resources. As relative indices, the MCI and NDCI yield values that must be converted to chl *a* concentration. This requires developing an empirical relationship between the index values and chl *a* values obtained through in situ sampling. Our objective was to derive and test a single empirical relationship for each algorithm spanning a broad suite of lakes across CONUS for each algorithm. Additionally, given errors often associated with atmospheric correction [34], we sought to test and compare results from three different radiance products for use in generating chl *a* estimates.

This effort demonstrates scaling of S2 chl *a* algorithms beyond individual lakes and watershed regions. This approach, using a single validation set to assess algorithm performance, is significant because it allows for a clear comparison of algorithms, which is not feasible using results from other efforts that focus on individual lakes, regions, and/or algorithms. The impact of this is three-fold. First, our investigation evaluated whether the use of S2 algorithms to estimate chl *a* is reproducible, a cornerstone metric of scientific reliability and an emerging priority for the earth science and ecological communities in particular [53,54]. Second, we assess the portability of these approaches. As efforts advance to apply land-based sensors such as S2 to inland waters, so too does the importance of evaluating existing algorithms in new settings and at different scales. This precedent has long been established by the ocean color community, specifically with use of the Ocean Chlorophyll algorithm [55,56]. Finally, we demonstrate the application of derived chl *a* measurements in an example waterbody. This study contributes toward advancing the maturity level of satellite-derived chlorophyll products [57], which may enable water quality managers to more broadly assess ecological status or condition across space and time, supporting the U.S. Clean Water Act reporting requirements.

## 2. Materials and Methods

**Figure 1.**
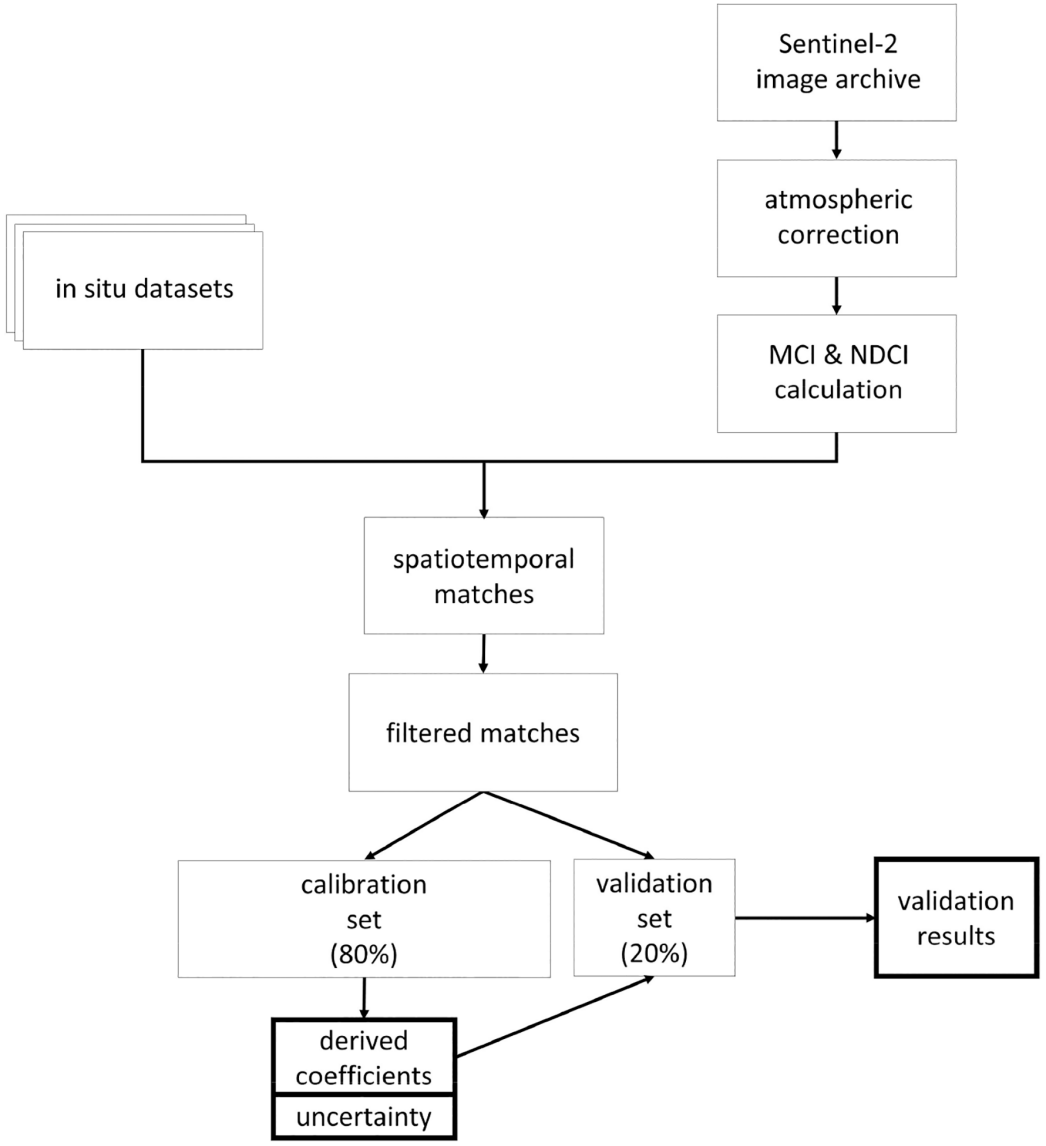
Diagram summarizing process for parameterizing the Sentinel-2 Maximum Chlorophyll Index (MCI) and Normalized Difference Chlorophyll Index (NDCI) and assess their performances.

We used a dataset containing in situ chl *a* values matched with S2 spectral data collected from the same locations and dates to parameterize the S2 MCI and NDCI and assess performance. The dataset was randomly split into a training set (80%) to develop the empirical relationship between MCI or NDCI and chl *a* and a testing set (20%) to assess performance of that relationship. The procedure is summarized in Figure 1 and explained further below. We also investigated a wide variety of factors that may influence error observed in algorithm retrievals.

### 2.1. In Situ Data

Our in situ chl *a* dataset was sourced from the U.S. Water Quality Portal (https://www.waterqualitydata.us [accessed on 1 May 2024]), USGS National Water Information System (https://nwis.waterdata.usgs.gov/nwis [accessed on 1 May 2024]), the Wisconsin Department of Natural Resources (DNR) data repository (https://data-wi-dnr.opendata.arcgis.com/ [accessed on 1 May 2024]), and Environment and Climate Change Canada (https://open.canada.ca/data/en/organization/ec [accessed on 1 May 2024]). We further added data from the AquaSat repository [58]. The dataset spans 2016–2020 and has previously been utilized in [48].

Several filtering criteria were applied to the in situ data. Only same-day matchups were included—that is, samples collected +/− 12 h from satellite overpass. Since satellite overpass typically occurs near midday, the 12 h threshold generally coincides with restricting to the same calendar date for in situ and satellite collection. Previous studies have demonstrated that such a temporal criterion is acceptable for matchup assessments over optically stable inland waters [37,59,60]. For coastal regions with tidal forcing though, we further restricted this time window to reduce any misrepresentation due to drastic changes in the water column. This combination strategy was chosen to balance the quantity and quality of our matchup statistics. Samples with an in situ collection time of midnight were excluded to remove any ambiguity of date. In situ sample depth was restricted to 2 m or less. Quality checks were performed to ensure no duplicates or replicates were present. Spatially, the analysis was confined to samples collected within lakes in the National Hydrography Dataset (NHD) Plus Version 2 shapefile. To prevent spectral contamination along the land–water interface, we also discarded samples collected in lakes but within 30 m of shore—a distance roughly equivalent to the diagonal of one 20 m pixel.

### 2.2. Satellite Data

Sentinel-2 MSI data were processed in NASA’s Sea-viewing Wide Field-of-view Sensor (SeaWiFS) Data Analysis System (SeaDAS: https://seadas.gsfc.nasa.gov [accessed on 1 May 2024]). SeaDAS was used to mathematically model and remove the contribution of various components in the atmosphere, including Rayleigh scattering, absorbing gases, and aerosols. SeaDAS has been shown to perform comparably to other open-source correction methods in inland waters for the red and near-infrared spectral bands used here [60,61]. SeaDAS was chosen over the Acolite processor since it provided consistently low-to-moderate error across all three wavelengths utilized in this study. Acolite exhibited substantially higher error than SeaDAS in the 740 nm band, while errors in the 664- and 705-nm bands were only slightly lower than for SeaDAS [60]. Our candidate matchup datasets included SeaDAS-processed MSI tiles uniformly resampled to 20 m resolution, representing a same-day overpass for in situ measurements, with a 3 × 3-pixel box centered on the closest geographic coordinates of the in situ measurement. Similar to the procedure explained in [29], our filtering criteria were based upon the standard procedure in [62]. Briefly, a matchup was discarded if any pixels in the 3 × 3-pixel box were quality flagged, and if four or more pixels were invalid, pixels suspected of contamination with sun glint and straylight were removed. We also removed matchup areas with coefficient of variation values (i.e., the ratio of standard deviation and mean in the green band) of > 50% for the nine pixels. The temporal mismatches were further tightened for dynamic aquatic ecosystems, such as the Chesapeake Bay, to +/− 30 min around the overpass time to minimize the associated uncertainties [63]. The median value over the 3 × 3-pixel box was then taken. In addition, ancillary parameters such as the sensor and solar viewing geometries and water vapor content were extracted.

Relative to band ratio algorithms, line height algorithms are considered less sensitive to uncertainties in atmospheric correction [64], particularly over inland waters. Some approaches have thus opted to use Rayleigh- and gas-corrected reflectance, hereafter referred to as Rayleigh-corrected reflectance (*ρ*_*s*_), in the interest of removing the effects of molecular (Rayleigh) scattering while avoiding aerosol correction, which is required to compute remote sensing reflectance (*R*_rs_), defined as the ratio of water-leaving radiance and downwelling irradiance just above the water surface. These include the Maximum Peak Height [52,65] and later instances of the Cyanobacteria Index [17]. Reflectance is often favored over radiance, as it enables comparisons across different latitudes and times of year [34]. For the sake of comparison, we report parameters and metrics derived for three separate reflectance products in the satellite processing chain: top-of-atmosphere reflectance (*ρ*_t_; no atmospheric correction), *ρ*_s_, and *R*_rs_ obtained by accounting for aerosol scattering and absorption using the band ratio of *ρ*_s_ in the 865 and 1613 nm bands [66].

### 2.3. Algorithms

**Figure 2.**
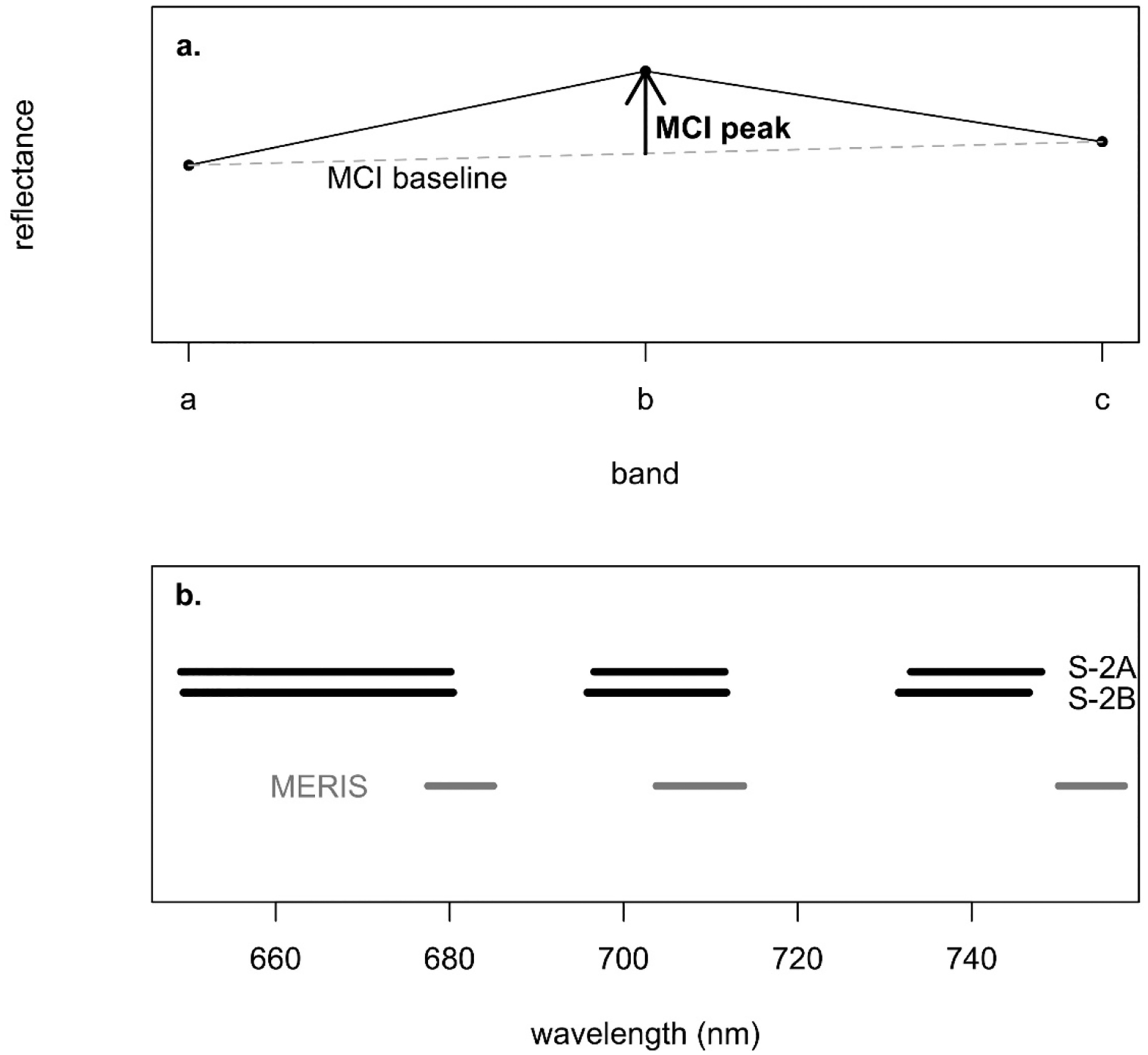
(**a**) Conceptual depiction of MCI calculation using example relative reflectance. A baseline is established between the baseline bands, a and c, and MCI is measured as the difference between reflectance of the peak band, b, and that baseline. (**b**) Visual representation of MERIS and S2 bands used to calculate MCI. MCI was originally developed based on MERIS data but is adapted here using the corresponding S2 bands.

The MCI was originally developed by [49] for the MERIS sensor onboard the Envisat satellite and subsequently also supported by Sentinel-3′s OLCI, which offers continuity in the required spectral bands. It leverages three bands in the red edge spectral range to measure a chl *a* signal, with two outer bands to establish a baseline and one central peak band. The original form was calculated using water-leaving radiance, but the same can be used for reflectance, as was used here for *ρ*_t_, *ρ*_s_, and *R*_rs_. The additional assessment carried out on diverse approaches to handle atmospheric correction is especially important for inland areas that contend with both an optically complex atmosphere and water bodies. The consideration of atmospheric correction will better ensure a more robust final chl *a* product. The difference between reflectance at the peak band and at the interpolated reflectance of the baseline at the same wavelength yields the MCI value (Equation (1), Figure 2a). Though S2 has a spectral configuration different to MERIS, it also has three bands in nearly the same waveband ranges as those used for MERIS MCI (Figure 2b, Table 1).

(1) $$MCI=R_{b}-R_{a}-\left[\frac{\lambda_{b}-\lambda_{a}}{\lambda_{c}-\lambda_{a}}\left(R_{c}-R_{a}\right)\right]$$

where *R* is reflectance, *λ* is the band wavelength, *a* is the left baseline band, *b* is the central peak band, and *c* is the right baseline band—665 nm, 705 nm, and 740 nm, respectively, for S2.

The presence of sediment in the form of mineral suspended particulate matter (MSPM) is known to interfere with the MCI algorithm, often resulting in elevated chl *a* retrievals and potential false positive bloom reports [67]. The spectra of MSPM-heavy waters diverge noticeably from those of clear waters. Ref. [67] presented a method to leverage this spectral characteristic, identifying samples with high MSPM concentration based on the slope of the MCI baseline (Figure 2a). That study suggested that MSPM contamination was likely when the baseline slope was more negative than −1.5 × 10^−4^ nm^−1^ for *R*_rs_ data. We removed samples below that threshold prior to calibration, validation, and subsequent analyses. Since no baseline slope thresholds for *ρ*_t_ and *ρ*_s_ were given in [67], we established relationships between baseline slope for each of those processing levels and *R*_rs_ to calculate surrogate baseline-slope thresholds specific to *ρ*_t_ and to *ρ*_s_.

Like the MCI, the NDCI (Equation (2)) was originally developed for MERIS and is calculated based on the magnitude of the chl *a* signal band around 705 nm, relative to a reference. Unlike the MCI, it uses only two bands: 665 nm and 709 nm for MERIS and OLCI (corresponding to 665 nm and 705 nm for S2). In the same manner as the normalized difference vegetation index (NDVI), the NDCI is calculated as the signal band reflectance (*R*_*b*_) less the reference band reflectance (*R*_*a*_), normalized by the sum of their reflectances, in an attempt to remove any uncertainty introduced by external factors such as atmospheric contribution or seasonality. Ref. [50] used remote sensing reflectance in their initial demonstration of the NDCI. Here, we tested the NDCI using *ρ*_t_, *ρ*_s_, and *R*_rs_.

(2) $$\text{NDCI}=\frac{R_{b}-R_{a}}{R_{b}+R_{a}}$$

where *R*_*a*_ is reference band reflectance at 665 nm and *R*_*b*_ is the signal reflectance around 705 nm.

### 2.4. Algorithm Calibration

Using a randomly selected 80% of in situ-satellite matchups as a training set and reserving the other 20% for subsequent testing, we constructed a linear model to convert spectral index values to chl *a*, fitting in situ chl *a* to the MCI or NDCI. Ranged Major Axis regression, a form of Model II regression, was used to construct the relationship, since both variables contained measurement error [68,69]; this was carried out using the *lmodel2* package in the R programming language [70]. As this form of regression is sensitive to outliers, we confirmed that none were present by calculating Mahalanobis distance in bivariate space for all chl *a* and spectral index values (MCI or NDCI). A chi-squared test was performed on Mahalanobis distance values, wherein a point would be considered an outlier if *p* < 0.001 [71]. The same training- and testing-data subsets were used for both algorithms and all satellite data processing levels to reduce potential differences stemming from data selection, allowing for more standard comparison between regression coefficients and validation metrics.

We employed a bootstrapping approach similar to [17] to derive slope (*β*_1_) and intercept (*β*_0_) regression parameters to construct the linear conversion model. Bootstrapping builds a series of randomly resampled datasets and uses each to fit a linear model [72]. Each dataset is built by sampling from the original dataset with replacement and is the same size as the original dataset, meaning that in each resampled dataset some observations may be selected more than once and some not at all. *β*_0_ and *β*_1_ values across all iterations are then averaged to yield the final coefficients. Bootstrapping offers an advantage over traditional single-instance model fitting because it enables the calculation of confidence intervals for each parameter and therefore confidence intervals for predictions. It also reduces the risk of overfitting to a particular data subset, as many model instances—each with its unique subset of the full dataset—are used to generate the final model. We bootstrapped 1000 iterations using the *boot* package in the R programming language [73] to generate each calibration regression, and selected bias-corrected, accelerated confidence intervals.

### 2.5. Algorithm Assessment

After calibrating the algorithms using 80% of the initial dataset, we used the remaining 20% of the data to test the relationships developed, comparing S2-derived chl *a* values to in situ chl *a* values. Conversion from MCI or NDCI to chl *a* resulted in some negative chl *a* values, which were removed. Note that MCI and/or NDCI values may be negative while still yielding positive chl *a* values, depending on linear-model intercept values fitted to the data; such observations were retained in the analysis. However, observations with negative chl *a*-predicted values were removed from the analysis; significance of negative values is discussed below and in Results.

To assess the performance of the MCI and NDCI algorithms, we plotted S2 chl *a* values against in situ chl *a* values to create scatter plots. Three error metrics were used to assess algorithm performance: mean absolute multiplicative error (MAE_mult_; Equation (3)), multiplicative bias (bias_mult_; Equation (4)), and mean absolute percentage error (MAPE; Equation (5)). Whereas traditional additive mean absolute error (MAE) provides the mean absolute difference between modeled and observed values, MAE_mult_ expresses error in terms of the factor by which modeled and observed values differ on average [74]. MAE_mult_ was selected over additive MAE since error in this study was proportional to in situ chl *a*, with higher error at higher in situ chl *a* values. A geometric mean was calculated because it more accurately expresses the central tendency of the data than an arithmetic mean when values are log-normally distributed, as they were here. As an example, a MAE_mult_ value of 1.5 indicates that modeled values and observed values are on average 50% different from each other. Bias was also reported in multiplicative terms. Whereas additive bias would report model underestimation as a negative value and overestimation as a positive one, multiplicative bias reports underestimation as a value less than 1 and overestimation as a value greater than 1; for example, a bias_mult_ value of 1.2 would indicate that modeled values are on average 1.2 times greater than observed values (20% greater), and a bias_mult_ value of 0.8 would indicate modeled values are on average 0.8 times observed values (20% less). MAPE is expressed in terms of percentage and thus was not transformed in the same manner as MAE_mult_ and bias_mult_.

(3) MAEmult=10^(∑i=1n|log10(Mi)−log10(Oi)|n)

(4) biasmult=10^(∑i=1nlog10(Mi)−log10(Oi)n)

(5) $$\text{MAPE}=\frac{\sum_{i=1}^{n}\frac{\left|M_{i}-O_{i}\right|}{M_{i}}}{n}$$

where *M* is modeled chl *a* for observation *i*, *O* is observed in situ chl *a* for observation *i*, and *n* is the sample size.

Algorithm performance can also be assessed by ability to correctly identify trophic state, an application likely to be relevant to stakeholders. We categorized in situ and single-pixel S2 chl *a* values by chl *a* ranges for each trophic state defined in the National Lakes Assessment [6], shown in Table 2. We then investigated the degree to which there was agreement using a confusion matrix and assessing accuracy.

### 2.6. Further Investigation and Application

In order to explore whether negative S2 chl *a* values may indicate low in situ chl *a* (potentially below algorithm detection sensitivity), we assessed whether in situ chl *a* measurements corresponding to negative S2 chl *a* values were different from in situ chl *a* values corresponding to positive S2 chl *a* values; this was carried out using the non-parametric Mann–Whitney U test [75,76]. These results were further summarized as effect size following the Glass [77] formulation of rank-biserial correlation (*r*_rb_). The effect size was classified according to Cohen [78], where 0.1 ≤ |*r*_rb_| < 0.3 indicates a small difference between datasets, 0.3 ≤ |*r*_rb_| < 0.5 indicates a moderate difference, and |*r*_rb_| ≥ 0.5 indicates a large difference. Large differences between datasets indicate results have practical significance, while small differences indicate limited practical applications.

Another nonparametric test, the Kruskal–Wallis test, was used to assess whether the collections of bootstrapped regression coefficients for *ρ*_t_, *ρ*_s_, and water-leaving reflectance (*ρ*_w_) were generated from the same population [79]. *ρ*_w_ was used in place of *R*_rs_ to enable a standardized comparison with *ρ*_t_ and *ρ*_s_, and was calculated as *R*_rs_ * π. These results were further summarized as effect size following the Kelley [80] formulation of epsilon-squared (_*ε*_^*2*^). The effect size was classified according to a modification of that of Cohen [78], where 0.01 ≤ |_*ε*_^*2*^| < 0.08 indicates a small difference between datasets, 0.08 ≤ |_*ε*_^*2*^| < 0.26 indicates a moderate difference, and |_*ε*_^*2*^| ≥ 0.26 indicates a large difference [81]. The Kruskal–Wallis test only determines if there is variation between regression coefficients of two or more of the processing levels. Therefore, when the Kruskal–Wallis test demonstrated that a large difference was present, post hoc pairwise Mann–Whitney U tests were performed to sequentially compare regression coefficients between each of the three processing levels.

To identify potential sources of algorithm error, we investigated several factors to search for relationships with chl *a* residuals. These factors included distance from shore, the temporal offset between in situ and satellite data collection, a suite of spectral information output by the SeaDAS processing software including individual spectral band reflectances and variables associated with atmospheric correction, and the suite of watershed physiographic variables included in the LakeCat database [82]. The analysis was performed by examining linear regression models fitting chl *a* error to each factor; magnitudes of *r*^2^ were used to assess strengths of relationships.

To demonstrate application of a S2 chl *a* product, we selected Jordan Lake, a eutrophic reservoir in central North Carolina (NC), to perform a brief case study. Jordan Lake serves as an important drinking water and recreational resource [83]. The State of North Carolina routinely monitors sites on the lake for several parameters, including chl *a* [84], and it has commissioned the development of a nutrient response model to understand the impact of nutrient loading on water quality [85]. Monitoring results are available from the NC Department of Environmental Quality and are used here to qualitatively assess performance and practicality of an S2 chl *a* product.

## 3. Results and Discussion

**Figure 3.**
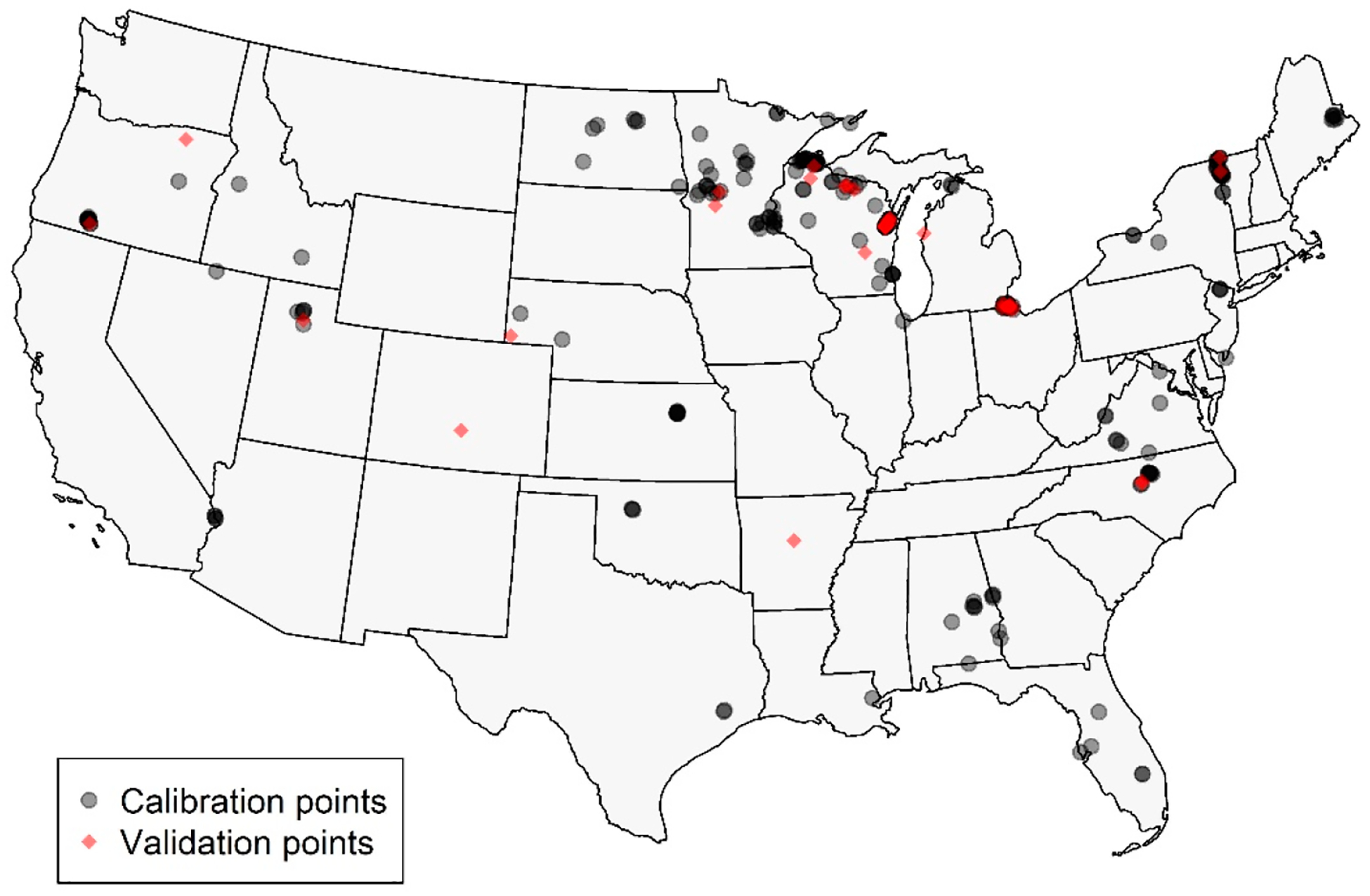
Map showing locations of calibration (gray) and validation (red) points across CONUS. Points are semi-transparent to show areas of higher density.

The dataset contained 300 in situ-to-satellite matchups. After filtering for MSPM using the baseline slope threshold, 294 observations representing 103 lakes remained for *ρ*_t_ and *ρ*_s_, and 296 for *R*_rs_; these observation counts are slightly different since the MSPM filtering is performed using reflectance values, which differed for each processing level. A total of 80% (*n* = 235 or 236) of these samples were used for calibration, and 20% (*n* = 59 or 60) were used for validation. In situ chl *a*, MCI, and NDCI values in each set are summarized for *ρ*_t_ in Table 3. The calibration and validation sets had similar distributions, though the validation set had slightly higher values for both in situ chl *a* and MCI. Geographically, calibration and validation points fell across CONUS, though they were highly concentrated in the upper Midwest and, to a lesser degree, in portions of the Eastern U.S. (Figure 3). The Western U.S. was sparsely represented, particularly by the validation dataset, which included only three samples from one lake. Portions of the West and Midwest were sparsely represented in both datasets; some of these areas are relatively arid and contain a lower density of lakes than much of CONUS, at least partially explaining the void, though the southern Midwest remains under-represented.

**Figure 4.**
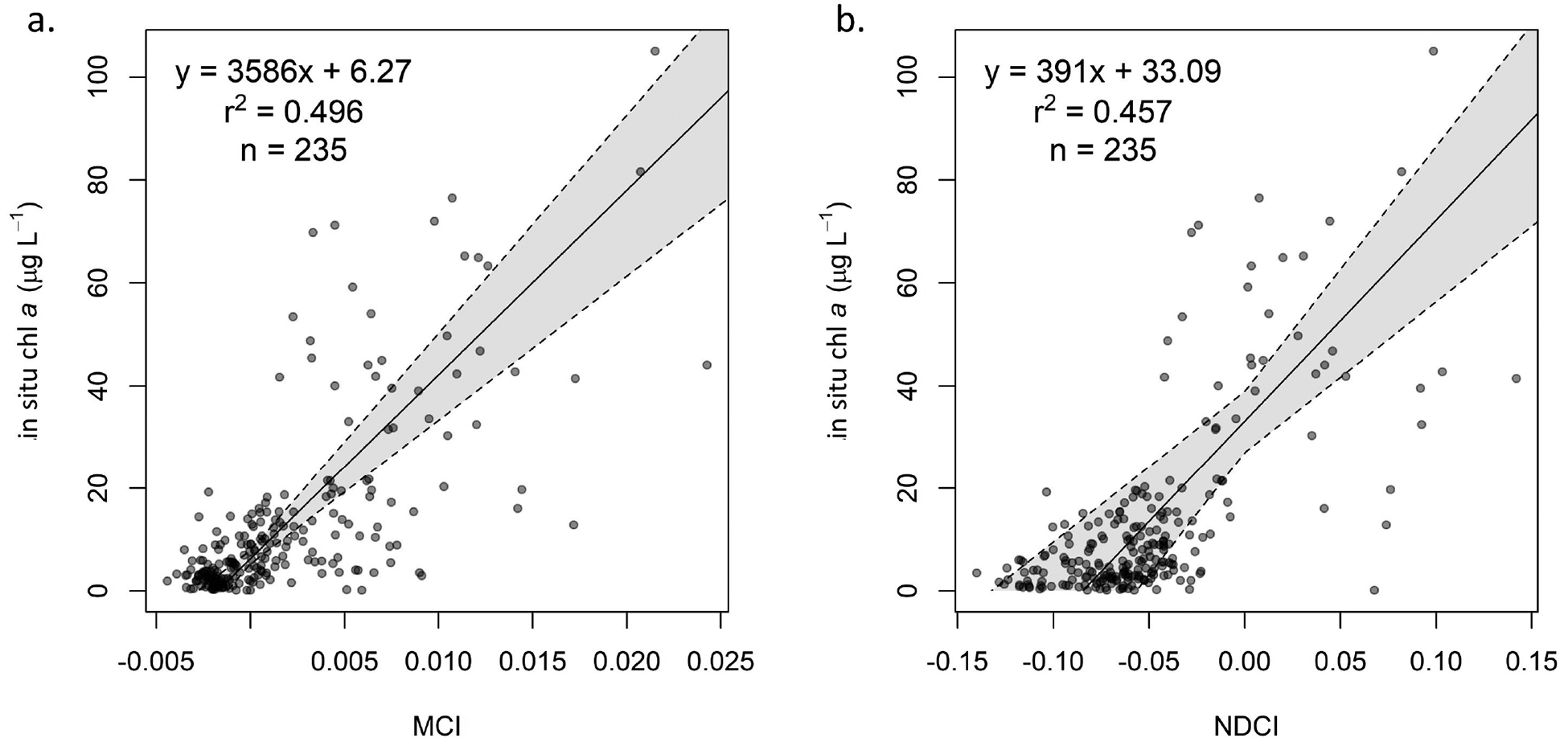
Calibration of (**a**) *ρ*_t_ MCI and (**b**) *ρ*_t_ NDCI, with 95% confidence interval based on bootstrapping distributions.

**Figure A1.**
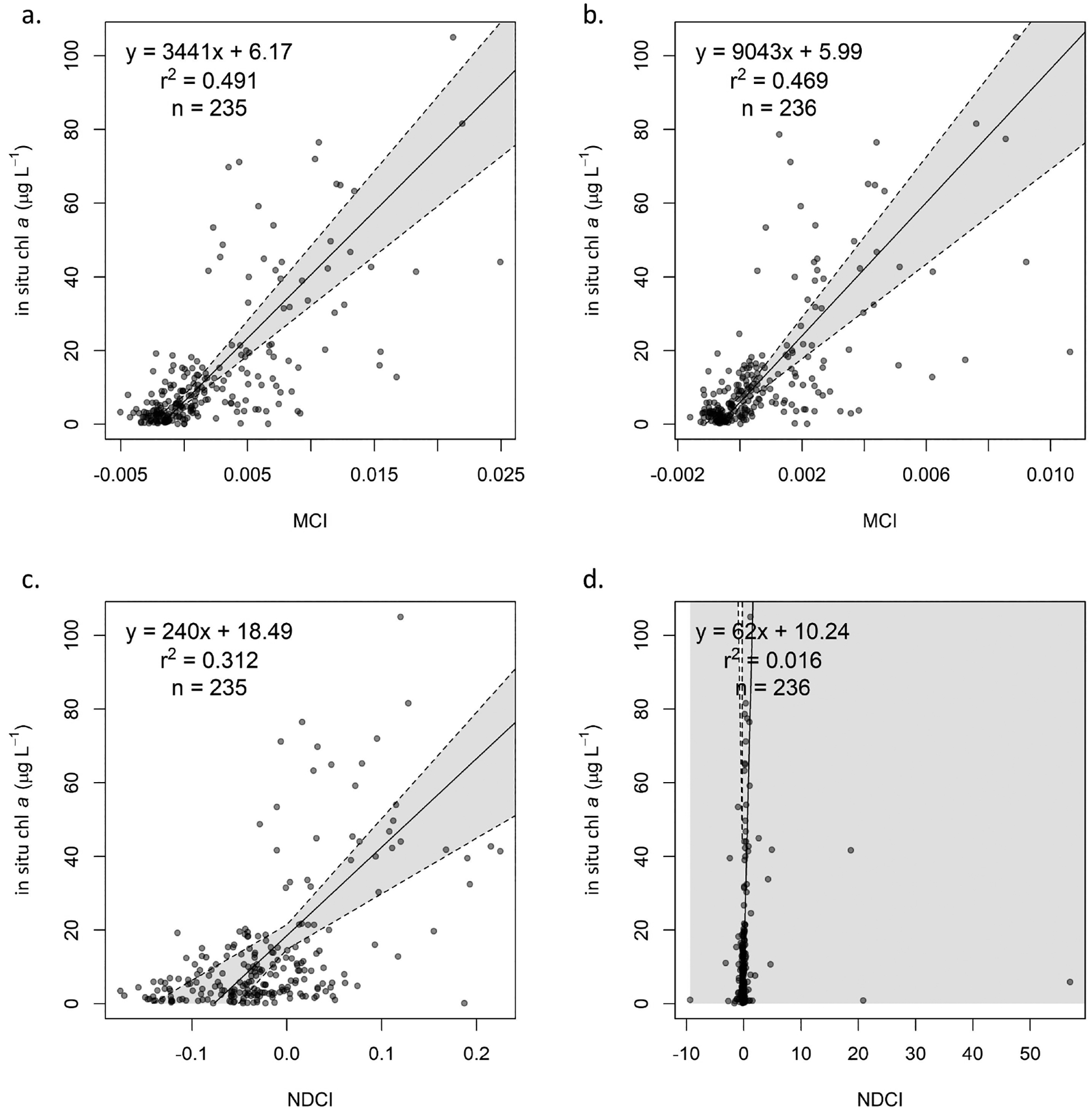
Calibration of MCI using and *ρ*_*s*_ (**a**) and $R_{rS}^{\Delta }$ (**b**) and NDCI using *ρ*_*s*_ (**c**) and $R_{rS}^{\Delta }$ (**d**), with dotted lines indicating 95% confidence intervals based on bootstrapping distributions.

The MCI and NDCI were calibrated and validated using the same dataset. For both algorithms, several models (exponential, polynomial) were explored for calibration, but a linear model consistently yielded the best fit according to *r*^2^ and visual inspection. With our dataset, the MCI outperformed the NDCI for all processing levels, with consistently lower MAE_mult_ and bias_mult_ values (Table 4, Figure 4). Since the NDCI is a ratio algorithm, extreme values are possible, particularly given uncertainties introduced by atmospheric correction; this is evident in the extremely high uncertainty, shown by wide confidence intervals, in the *R*_rs_ calibration (Figure A1d). For both MCI and NDCI, *ρ*_t_ had the lowest MAE_mult_ values compared to *ρ*_s_ and *R*_rs_, though for MCI, *ρ*_s_ exhibited only slightly higher MAE_mult_ than *ρ*_t_. Further analysis focused on *ρ*_t_-derived MCI since it tended to yield the most reliable results; information for *ρ*_s_ and *R*_rs_ can be found in Appendix A.

### 3.1. Calibration

For the MCI, regression coefficients for the three reflectance products are reported in Table 4. All three relationships showed similar goodness of fit, with *r*^*2*^ values near 0.5. Note that, though *β*_0_ values for the three products are similar, *β*_1_ for *R*_rs_ is substantially larger than for *ρ*_t_ and *ρ*_s_. Though aerosol correction may account for some of the difference between *R*_rs_ and *ρ*_s_, the main reason for the larger *β*_1_ value *R*_rs_ is that reflectance values are divided by π when converted from water-leaving reflectance (*ρ*_w_, the fully atmospherically corrected analogue of *ρ*_s_). Thus, since coefficients for *R*_rs_ cannot be compared to *ρ*_t_ and *ρ*_s_, we converted *R*_rs_ to *ρ*_w_ for the purpose of—and solely for the purpose of—determining whether the various atmospheric processing steps strongly influenced the empirical conversion relationships.

Results of the Kruskal–Wallis test and associated epsilon-squared demonstrated that differences in *β*_1_ for *ρ*_t_, *ρ*_s_, and *ρ*_w_ (the analog of *R*_rs_ used for standardized comparison with the other processing levels) were moderate (_*χ*_^2^ = 356; DF = 2; |_*ε*_^*2*^| = 0.12) and differences in *β*_0_ were large (*χ*^2^ = 1661; DF = 2; |*ε*^*2*^| = 0.55). For both coefficients, these differences were driven by smaller coefficients for *ρ*_w_ compared to both *ρ*_t_ and *ρ*_s_, though for *β*_1_, differences between *ρ*_s_, and *ρ*_w_ were small. Comparing coefficients between *ρ*_t_ and *ρ*_s_, differences were small for *β*_1_ and negligible for *β*_0_, indicating little difference between the conversions for the two products. For *β*_0_, *ρ*_t_, *ρ*_s_, and *ρ*_w_ medians were 6.3, 6.2, and 4.3, respectively, with the post hoc Mann–Whitney U test indicating large differences between both *ρ*_t_ and *ρ*_w_ (*U* = 960,138, *N* = 2000, |*r*_rb_| = 0.92) and *ρ*_s_ and *ρ*_w_ (*U* = 949,751, *N* = 2000, |*r*_rb_| = 0.90). Note that *r*^*2*^ for *ρ*_w_ (0.32) was much lower than that for *R*_rs_ (0.47), suggesting that the relationship for *ρ*_w_ exhibited lower fit than that for *R*_rs_.

Table 4 also provides regression coefficients for the NDCI calibrations for *ρ*_t_ (Figure 4b), *ρ*_s_ (Figure A1c), and *R*_rs_ (Figure A1d). Generally, confidence intervals were wider than for MCI, particularly for *R*_rs_ (Table 4). Additionally, the NDCI appears more sensitive to selection of atmospheric processing level; while MCI exhibited similar coefficients for *ρ*_t_ and *ρ*_s_, NDCI yielded coefficients that were notably different and with non-overlapping confidence intervals.

### 3.2. Validation

**Figure 5.**
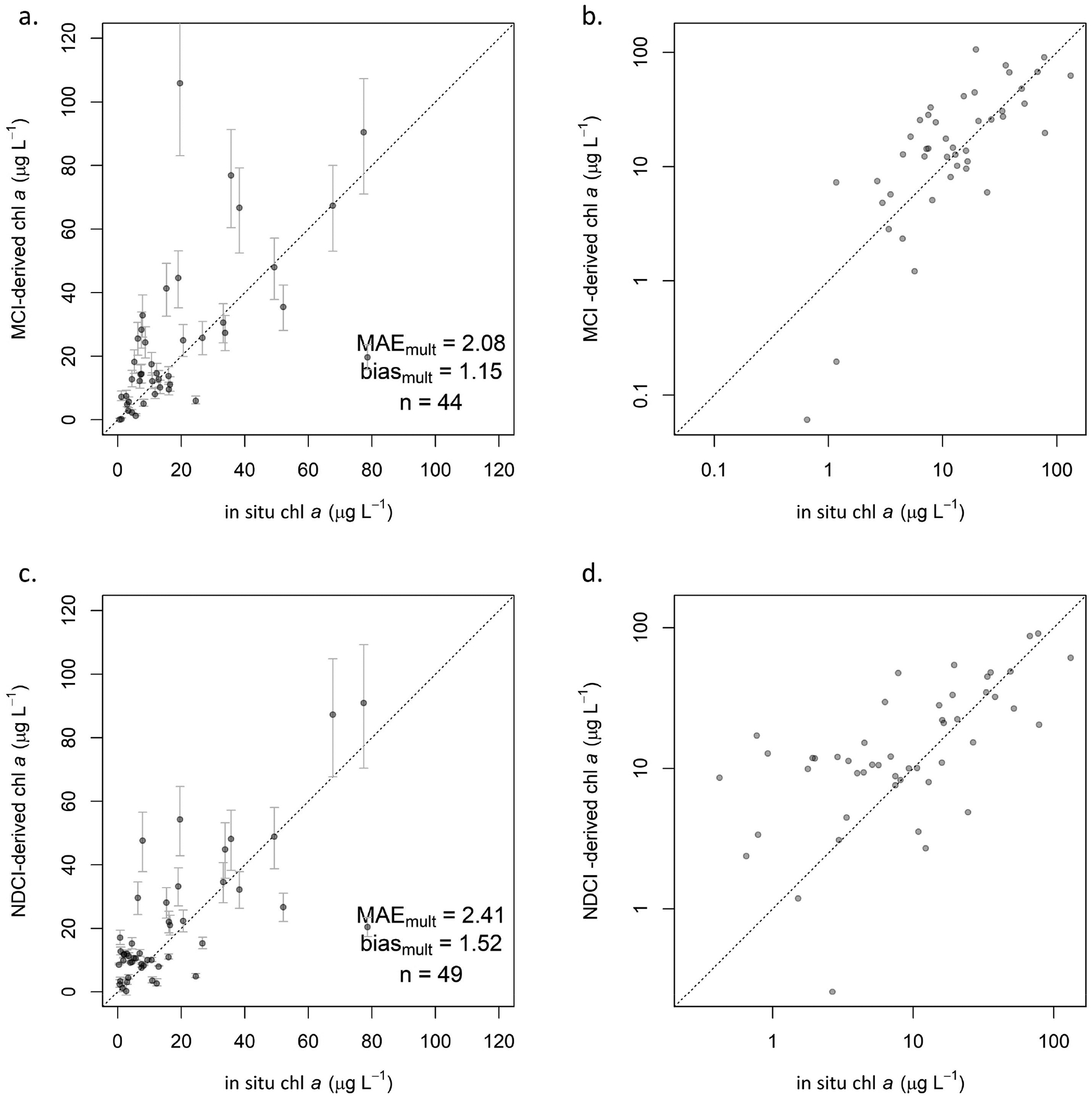
Validation scatterplots showing S2-derived chl *a* on *y*-axis and reference in situ chl *a* on *x*-axis: (**a**) *ρ*_t_ MCI in linear space, including 95% confidence intervals and (**b**) with both axes log-transformed, and (**c**) *ρ*_*t*_ NDCI in linear space, including 95% confidence intervals and (**d**) with both axes log-transformed. Dotted lines indicate 1:1 relationship.

**Figure A2.**
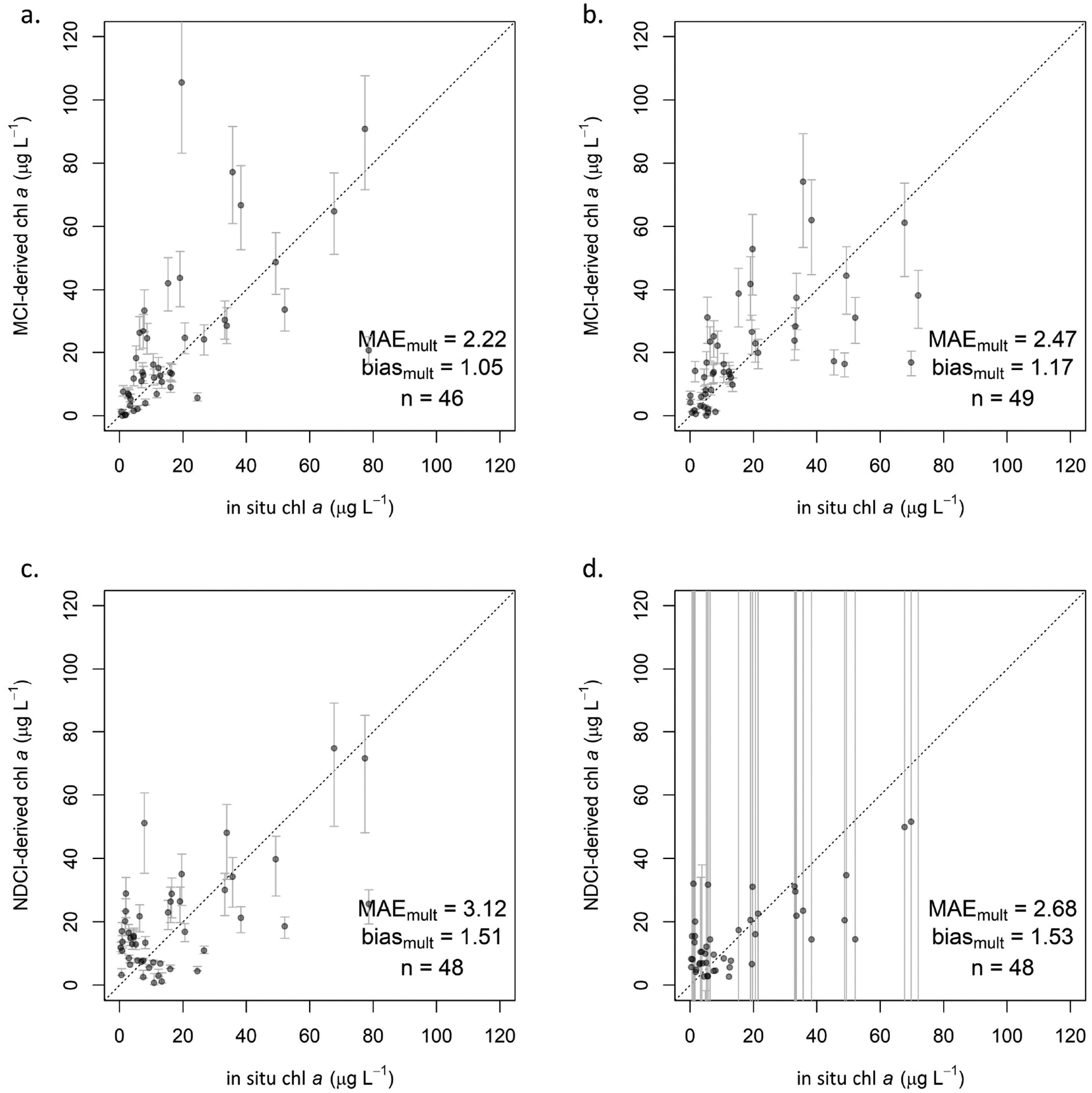
Validation of MCI using *ρ*_*s*_ (**a**) and Rrs (**b**) and NDCI using *ρ*_*s*_ (**c**) and Rrs (**d**), with 95% confidence intervals (extending beyond plot extent in (**d**)) and dotted line indicating 1:1 relationship.

Using the relationships established in the calibration step, chl *a* predictions were derived from MCI and NDCI values in the validation set for each processing level. For MCI, all three processing levels generally performed similarly, as indicated by validation statistics (Table 4). *ρ*_t_ (Figure 5a,b) exhibited the lowest MAE_mult_ of 2.08 by a small margin, though *ρ*_s_ (Figure A2a) showed the smallest bias_mult_ of 1.05. *R*_rs_ (Figure A2b) showed the highest MAE_mult_ of 2.47 and a notably higher MAPE of 307%. All three processing levels showed slight positive biases (bias_mult_ > 1), indicating overestimation, but not substantially so.

Error rates for NDCI were consistently higher than for MCI. Bias_mult_ for all processing levels was near 1.5, indicating a consistent overestimation. *ρ*_t_ exhibited lower MAE_mult_ (2.41; Figure 5c,d) than did *ρ*_s_ (3.12; Figure A2c), as with MCI, but unlike with MCI, *R*_rs_ (2.68; Figure A2d) was intermediate between *ρ*_t_ and *ρ*_s_. However, *R*_rs_ yielded erratic calibration results (Figure A1d), with extremely broad confidence intervals that included negative values for both *β*_1_ and *β*_0_. A majority of NDCI values for all reflectance products fell between −0.2 and 0.2, but *R*_rs_ yielded several values well outside that range, including 26 of 300 in the full dataset that were outside of −1 < NDCI < 1. Though these values were extreme relative to the other reflectance products, they were not identified as outliers, likely because there were several of them. Further investigation indicated that the cause of these extreme values was negative reflectance values, which only existed in *R*_rs_. Such extreme NDCI values were observed to occur when one band (665 nm or 705 nm) had a negative reflectance, and the other had a positive reflectance—even if the reflectance values themselves were not extreme, i.e., barely positive or negative. This was observed to occur in both directions, resulting in highly positive NDCI values when *R*_705_ > *R*_665_, and highly negative ones when *R*_705_ < *R*_665_. Based on these results, caution is advised when considering the use of *R*_rs_ for NDCI, although the algorithm was developed using *R*_rs_. Any uncertainty in atmospheric contribution—whether from correction or from lack thereof—is exacerbated in the algorithm’s calculation; assuming the atmospheric contribution is similar at the 665 nm and 705 nm bands, it is effectively removed in the numerator by subtracting the two bands’ reflectance values but doubled in the denominator by adding those values.

**Figure 6.**
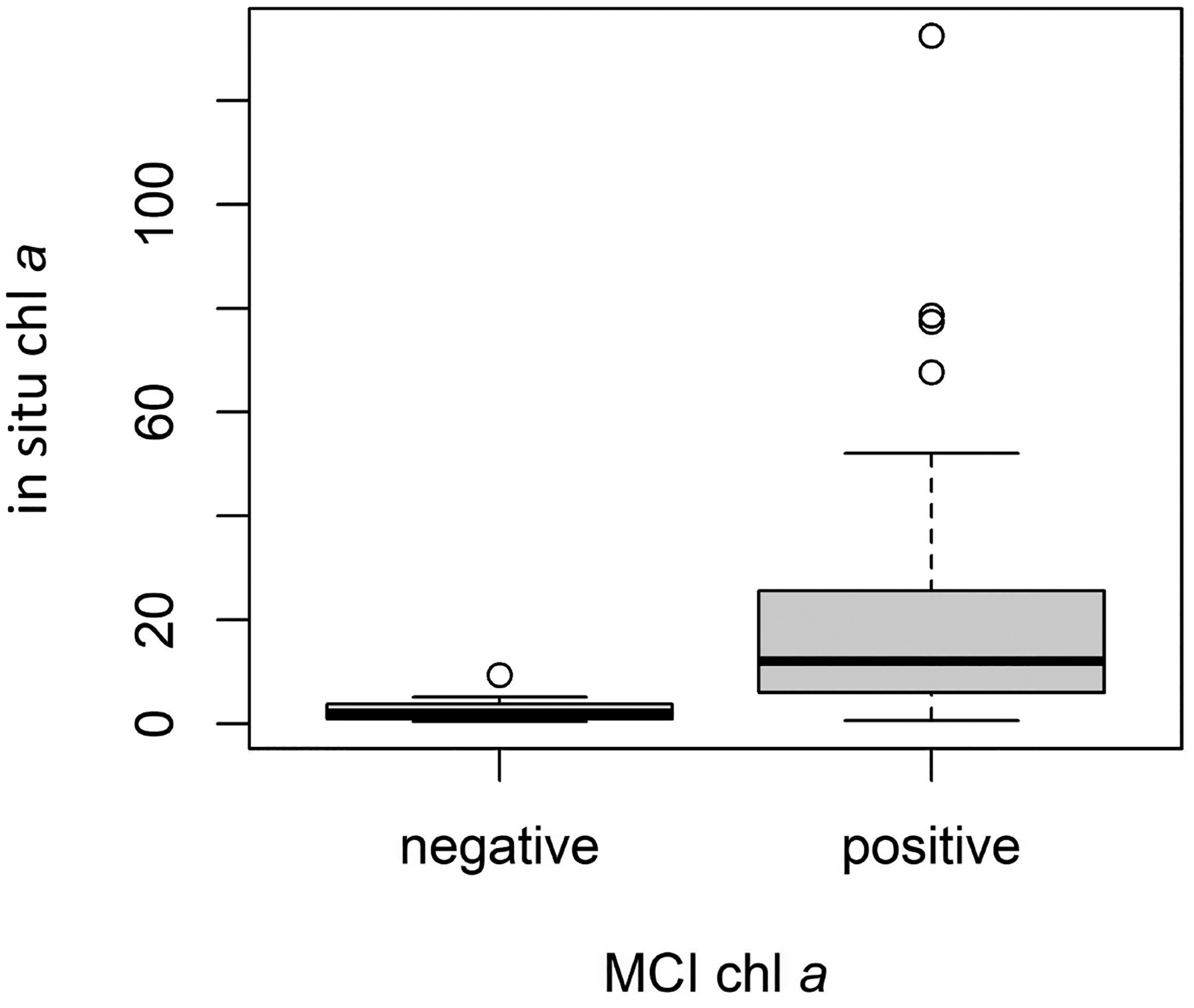
Comparison of in situ chl *a* values for negative (*n* = 15) and positive (*n* = 44) *ρ*_t_ MCI-derived chl *a* instances. Dark lines indicate medians, boxes extend from lower to upper quartiles and indicate the interquartile range (IQR), whiskers extend beyond lower and upper quartiles by 1.5 × IQR, and points indicate any values outside 1.5 × IQR.

Conversion from MCI and NDCI to chl *a* resulted in several instances of negative chl *a* values. Though these values are not inherently erroneous, we excluded them from the analysis since they were assumed to indicate low response, potentially reflecting algorithm detection limits. For MCI, *ρ*_t_ yielded 15 negative values, *ρ*_s_ yielded 13, and *R*_rs_ yielded 11, partly explaining the different sample sizes for each validation, with differences in the MSPM filtering explaining the remaining differences. Notably, in situ chl *a* values for samples removed due to negative S2 MCI chl *a* values tended to be substantially lower than those retained in the validation dataset. For *ρ*_t_, in situ chl *a* values for negative S2 chl *a* instances (*n* = 15) ranged from 0.4 to 9.3 μg L^−1^, with a median of 1.9 and a mean of 2.7 μg L^−1^, while positive instances (*n* = 44) ranged from 0.65 to 132.4 μg L^−1^, with a median of 12.0 and a mean of 21.4 μg L^−1^ (Figure 6). Results of the Mann–Whitney U test and associated rank-biserial correlation demonstrated that in situ chl *a* measurements were substantially lower for negative S2 chl *a* values than for positive S2 chl *a* values, indicating a large difference between the two groups (Figure 6; *U* = 590, *N* = 59, |*r*_rb_| = 0.79). Negative S2 chl *a* instances tended to be lower and much closer to zero, supporting the notion that they may be treated as non-detections. Furthermore, the distribution of in situ chl *a* values for the set of negative S2 chl *a* values indicates the point at which the MCI can no longer reliably discern the presence of chl *a* from its absence, suggesting a rough detection limit around or below 10 μg L^−1^, though the algorithm still provided useful information below this threshold in many cases (see Figure 5b). This finding coincides with the MCI detection limit of 10 μg L^−1^ suggested by [86].

**Figure A3.**
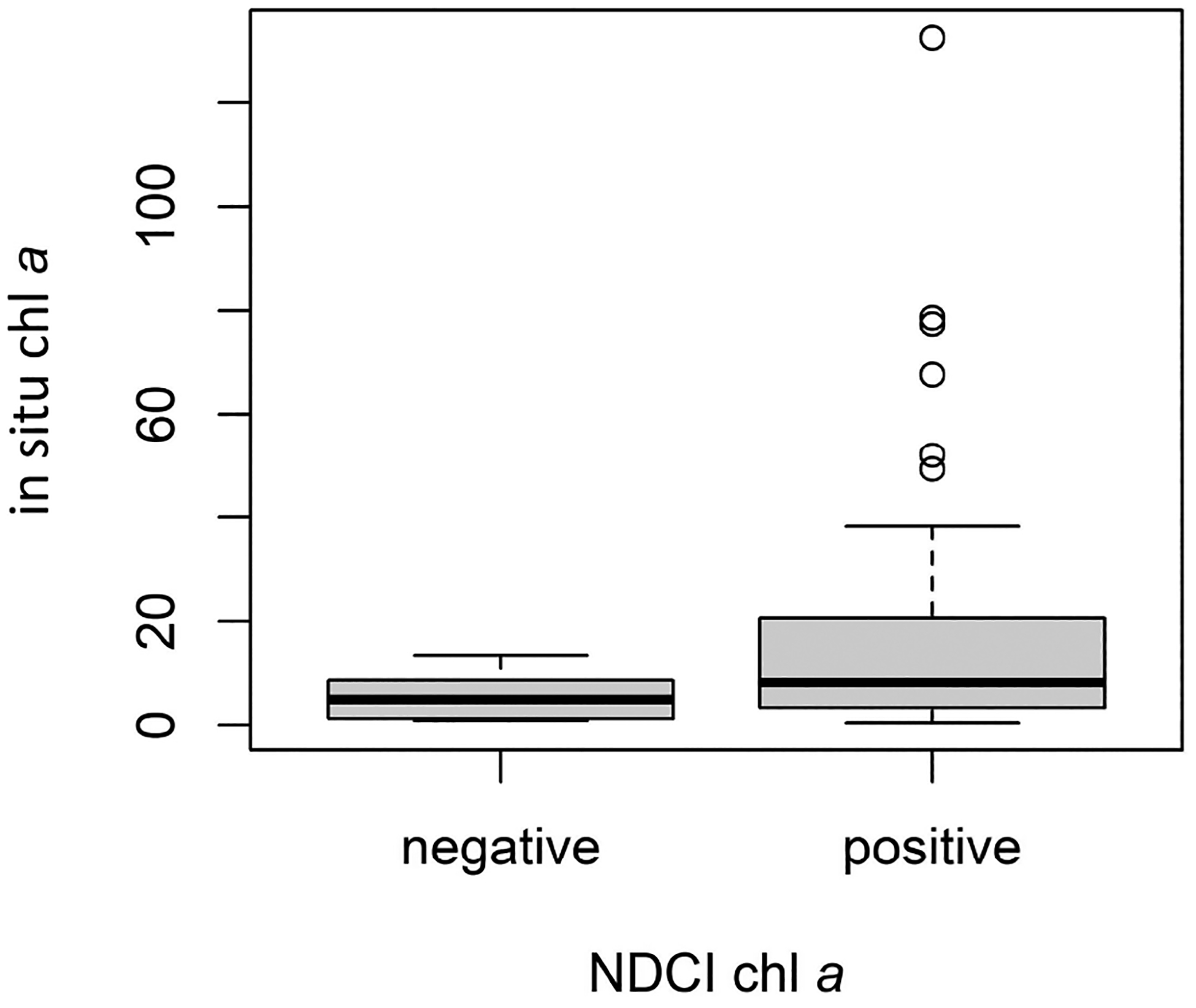
Comparison of in situ chl *a* values for negative (*n* = 10) and positive (*n* = 49) *ρ*_t_ NDCI-derived chl *a* instances. Dark lines indicate medians, boxes extend from lower to upper quartiles and indicate the interquartile range (IQR), whiskers extend beyond lower and upper quartiles by 1.5 × IQR, and points indicate any values outside 1.5 × IQR.

For NDCI *ρ*_t_, negative and positive S2 chl *a* instances were less separable (Figure A3). In situ chl *a* values for negative S2 chl *a* instances (*n* = 10) ranged from 0.8 to 19.2 μg L^−1^, with a median of 3.5 and a mean of 4.5 μg L^−1^, while positive instances (*n* = 49) ranged from 0.42 to 132.4 μg L^−1^, with a median of 8.1 and a mean of 18.8 μg L^−1^. Results of the Mann–Whitney U test and associated rank-biserial correlation demonstrated that in situ chl *a* measurements were only moderately lower for negative S2 chl *a* values than for positive S2 chl *a* values (Figure A3; *U* = 324, *N* = 59, |*r*_rb_| = 0.32). This suggests that the detection limit for NDCI may be higher than for MCI—observations with higher in situ chl *a* concentrations were assigned negative S2 chl *a* values, i.e., classified as non-detection. Further studies with larger datasets are required to provide additional insights and to report more quantitative detection limits.

### 3.3. Trophic State

The ability of the S2 MCI to correctly identify trophic state was assessed using a confusion matrix (Table 5) with the number of samples correctly identified in bold, the number omitted, and the number committed in each trophic state. For *ρ*_t_, S2 MCI chl *a* correctly identified in situ trophic state 66% of the time (29 of 44 observations) and was within one class of correct for all observations but one; accuracy was 67% for *ρ*_s_ and 59% for *R*_rs_. A simplified binary classification that combined the oligotrophic–mesotrophic and eutrophic–hypereutrophic ranges showed increased accuracy of 82% (36 of 44 observations) for *ρ*_t_, 83% for *ρ*_s_, and 84% for *R*_rs_. NDCI trophic state classification accuracies were lower than for MCI (Table A1). S2 NDCI chl *a* correctly identified the in situ trophic state 47% at the time (23 of 49 observations) for *ρ*_t_, 35% for *ρ*_t_, and 40% for *R*_rs_. Binary non-eutrophic/eutrophic classifications yielded an overall accuracy of 63% (31 of 49 observations) for *ρ*_t_, 48% for *ρ*_s_, and 60% for *R*_rs_.

Investigating error in each trophic state indicates how algorithm performance varies across the range of chl *a* values. Multiplicative error metrics (MAE_mult_ and bias_mult_) were used throughout this study when assessing error in aggregate since that error was proportional to chl *a* values. However, additive metrics (MAE_add_ and bias_add_) may also be useful when investigating smaller ranges such as trophic state classes, across which the magnitude of error changes less dramatically. Considering *ρ*_*t*_, for both MCI (Table 6) and NDCI (Table A2) MAE_add_ tended to increase with increasing trophic state—aligning with the notion that error was greater at higher chl *a* values. However, MAE_mult_ decreased with each successive trophic state class. Performance at most trophic state classes were similar for MCI and NDCI; the better overall performance of MCI could likely be attributed to the oligotrophic class, which had the highest MAE_mult_ of all classes for both algorithms but contained more points for NDCI (*n* = 8) than for MCI (*n* = 3)—thus pulling the overall MAE_mult_ for NDCI error up. NDCI exhibited a highly positive bias (overestimation) in the oligotrophic class, whereas MCI did not. Both algorithms exhibited slight negative bias (underestimation) in the hypereutrophic class.

Poor performance at low values may be explained by the spectral configuration of both algorithms. Though both are measuring the reflectance peak at 705 nm, the maximum reflectance of the chl *a* spectral signal tends to be at shorter wavelengths, particularly at low concentrations [14]; the 705 nm band would be measuring a lower reflectance on the peak’s right-hand shoulder. As concentration increases, the peak shifts to longer wavelengths and is better captured by the 705 nm band. This phenomenon of diminished signal at low values introduces uncertainty into both the calibration and validation results, particularly because of the high occurrence of values at lower concentrations. Poorer performance by the NDCI at low values may also be attributable to the calculation method—division versus subtraction—or, potentially, to the consideration of an additional spectral band in the MCI.

### 3.4. Comparison with Other Studies

Several other studies have tested S2 chl *a* algorithms, including the MCI, NDCI, and similar approaches, mostly on limited datasets. Many report *r*^2^ values on the relationship between an optical index and in situ chl *a* as opposed to converting the algorithm output to chl *a*. The relationship between MCI S2 and in situ chl *a* generated for the MCI calibration in our study yielded *r*^2^ = 0.50. Note that *r*^2^ should only be used to assess goodness of fit of a relationship (i.e., calibrate), not to assess accuracy of an algorithm (i.e., validate). To compare S2 chl *a* estimates to reference in situ data, the preferred metrics are MAE, bias, and MAPE [74].

Past work has demonstrated the application of the MCI. Ansper and Alikas [35] fitted MCI to in situ data using the S2 Level 1C (L1C—equivalent to *ρ*_t_) on chl *a* for 12 data points from a collection of Estonian lakes ranging from 15 to 35 μg L^−1^, and reported *β*_1_ = 870.8 and *β*_0_ = 25.3—quite different than those that we derived, and indicating less response to changing chl *a* values—with a fairly poor fit (*r*^2^ = 0.25). Toming et al. [87] employed an algorithm that equates almost exactly to the MCI, with only a minor difference in calculation, in 11 Estonian lakes with in situ chl *a* data spanning 3.6 to 72.9 μg L^−1^, a range similar to ours. They fit algorithm index values to in situ chl *a* using bottom-of-atmosphere (BOA—equivalent to *ρ*_w_ or *R*_rs_ × π) data, deriving *β*_1_ = 2231 and *β*_0_ = 12.7 (*r*^2^ = 0.80). Ref. [43] investigated several algorithms including MCI, using 273 observations with in situ chl *a* ranging from 0.00 to 120.99 μg L^−1^. Though they reported high scatter for the MCI, that algorithm exhibited the lowest MAPE (70.1%) and among the lowest MAE values (5.74 μg L^−1^) of nine algorithms tested. Likewise, Pirasteh et al. [88] reported that, of several algorithms tested for S2, MCI performed best (*r*^2^ = 0.92) for chl *a* > 8 μg L^−1^. Similarly, studies have tested the NDCI using S2. Ref. [45] used atmospherically corrected S2 data—presumably *ρ*_w_—to fit NDCI to in situ chl *a* with 28 points in a single lake (*β*_1_ = 179.18, *β*_0_ = 10.82, *r*^2^ = 0.710). They calculated root mean squared error (RMSE) to be 4.70 μg L^−1^, an independent set of 28 additional points. Ref. [46] tested the NDCI with S2 *R*_rs_ data using an in situ chl *a* range of 1.89–70.20 μg L^−1^ and the calibration coefficients provided in the original NDCI development—which used MERIS, not S2. They observed an RMSE value of 14 μg L^−1^ and a strong bias of −9.28 μg L^−1^.

Several studies have utilized other algorithms to estimate chl *a* using S2. Ref. [89] investigated several ratio algorithms using linear, exponential, and logarithmic relationships in a single lake, reporting a broad range of success (*r*^2^ from 0.00 to 0.68), though in situ chl *a* values spanned from only 1.6 to 6 μg L^−1^, all below the potential detection limit of 10 μg L^−1^ identified by [86] and corroborated in our findings. Likewise, [90] validated several chl *a* algorithms across a low range (0 to 9 μg L^−1^, again below the suggested detection threshold of MCI) in two Mediterranean bays, with the best performing algorithms yielding MAE_mult_ between 0.41 and 0.71. Ref. [91] compared results from a three-band algorithm to 92 in situ chl *a* samples spanning time across a single lake, ranging from 4.5 to 209 μg L^−1^ with an average of 97 μg L^−1^—a distribution generally higher than ours. They reported a MAPE value of 9.6%, quite low compared to the value we reported. Ref. [45] employed an ensemble of three algorithms, all post-atmospheric correction, to estimate chl *a* in a single lake. With 28 in situ chl *a* data points spanning from 8 to 61 μg L^−1^ and averaging 27 μg L^−1^, they compared individual algorithms, an optimally weighted ensemble, and a spectral space partition guided ensemble. Individual algorithms yielded RMSE between 4.5 and 4.7 μg L^−1^, while the optimally weighted ensemble improved RMSE to 4.1 μg L^−1^, and the partition guided ensemble showed further improvement to 3.57 μg L^−1^. These studies examined one or two waterbodies, in some cases with lower error than that observed in our study. That is likely due to the limited optical variability in individual systems versus that across several systems. In a broader effort, [48] retrieved chl *a* values using a machine learning approach—a mixture density network—on over 800 S2 observations coinciding with in situ chl *a* samples globally, with a focus on North America. That study reported a performance similar to that reported here, with an uncertainty of 80% (comparable to MAE_mult_ of 1.80—slightly lower than in our study) and bias of 41% (comparable to bias_mult_ of 1.41—slightly higher than in our study). Like our study, [48] demonstrated the application of S2 on a broad scale, but the current work provides a simple method that may be more accessible for stakeholder adoption.

Efforts to retrieve chl *a* with MERIS using MCI, NDCI, and similar approaches indicate that performance is similar to that of S2, or perhaps slightly better. In early work applying the MCI in North America, [86] fit relationships between MCI derived from in situ radiometric data (*ρ*_w_) and in situ chl *a* values in Lake Ontario (*β*_1_ = 5000, *β*_0_ = 6, *r*^2^ = 0.78), Lake Erie (*β*_1_ = 2500, *β*_0_ = 5.25, *r*^2^ = 0.70), and Lake of the Woods, located along the U.S.–Canada border (non-linear, *r*^2^ = 0.91). These calibrations may have shown stronger relationships than ours because they were performed on individual lakes and are developed using spectral information collected directly above the water column and not interacting with the atmosphere or affected by atmospheric correction. The study introducing the NDCI with MERIS assessed that algorithm using 14 field validation points with in situ chl *a* ranging from 0.9 to 28.1 μg L^−1^ in two marine bays, yielding RMSE = 2.37 μg L^−1^ [50]. Ref. [17] developed an empirical relationship between 348 concurrent in situ chl *a* and the MERIS-derived Cyanobacteria Index measurements, reporting MAE_mult_ of 1.62 and bias_mult_ of 1.11. The in situ chl *a* values in that study tended to be much greater than those herein, with a range of 0.5 to 832 μg L^−1^, a mean of 71.2 μg L^−1^, and a median of 45 μg L^−1^.

When using in situ data as a reference against which to compare satellite-derived values, it is important to note that in situ data also have error, which is often not reported. For chl *a*, prior studies have found in situ sampling methods have an average error of 39%, with error as high as 68% [92,93]. The in situ data used here were acquired via a variety of collection and analysis methods by several different organizations, further contributing to inconsistencies between sample points and variability in in situ error. Sub-pixel variability may introduce additional inconsistencies; though this is much less of a concern for S2 than it is for S3, for example, a fair degree of heterogeneity in chl *a* concentration is possible across a 20 m space. There is no way of definitively assessing the error of the in situ data used in this study, nor whether it tends to be biased in one direction or the other. Still, comparing the 101% MAPE observed in this study to in situ measurement error reported in prior work suggests that (a) S2 chl *a* measurement error may not be substantially greater than in situ measurement error, and (b) a large portion of error observed in this study may be due to in situ error.

### 3.5. Analysis of Error

**Figure 7.**
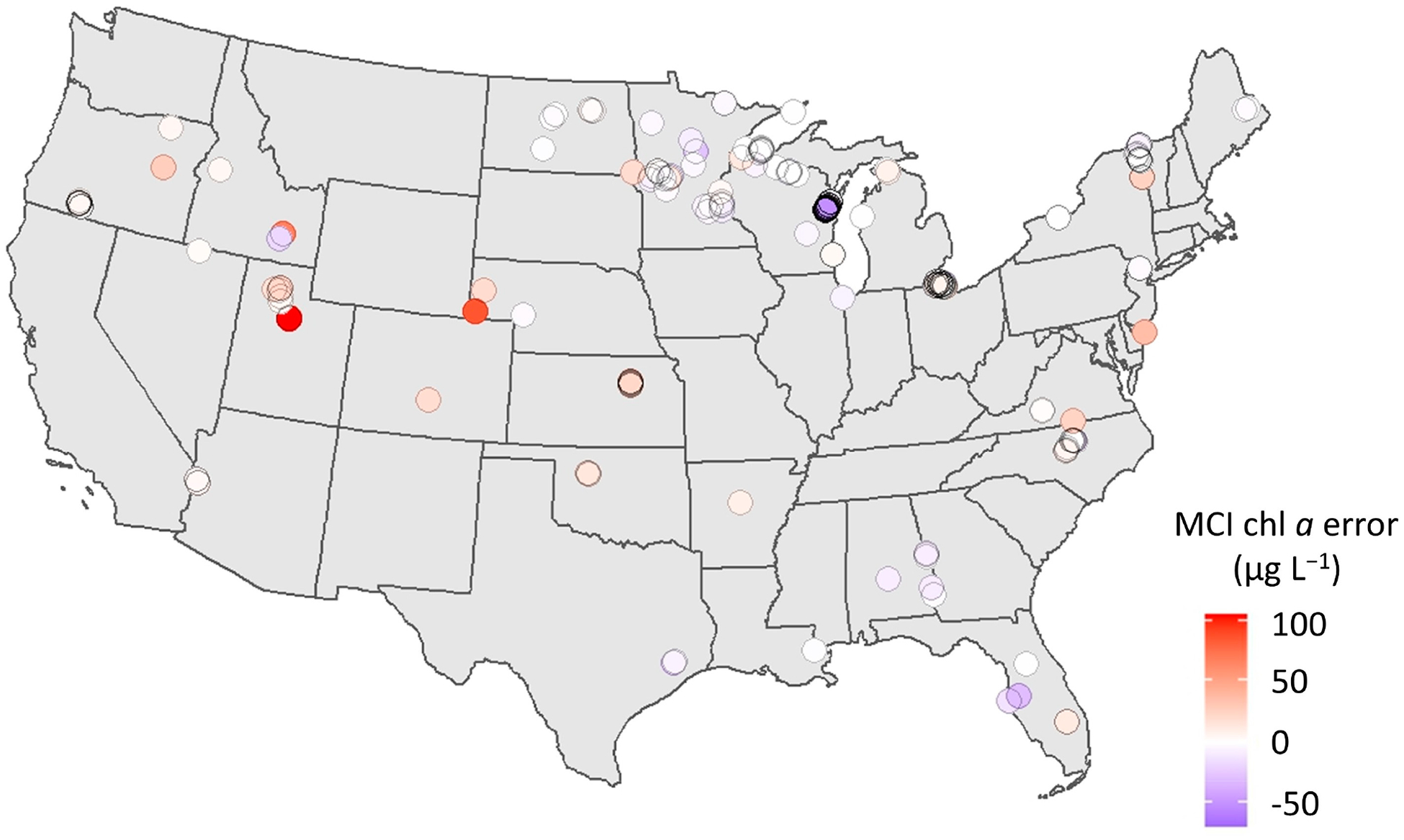
Map showing direction and magnitude of chl *a* error (μg L^−1^) for each point across CONUS, including both calibration and validation matchups. Red indicates overprediction by S2 MCI (positive error); blue indicates underprediction (negative error).

To understand the limitations of the MCI and which conditions warrant caution when applying the algorithm, it is useful to search for patterns in error. This exercise may also facilitate improvements to the algorithm, including the flagging of certain conditions and, potentially, the development of separate parameterizations for different conditions or regions. Error was first investigated spatially across CONUS (Figure 7) using the full dataset, including both calibration and validation data. Though distribution of sample points is limited in space, one pattern is observable: overestimation in the Western U.S. Nearly all samples west of Minnesota exhibit positive biases, while samples in the Eastern U.S. exhibit both positive and negative bias.

To attempt to explain the spatial bias of error and to identify potential confounding factors, we explored relationships between error and various environmental variables using linear regression. Generally, none of the factors showed a relationship with S2 chl *a* residuals with *r*^2^ greater than 0.25, with two exceptions. In situ dissolved organic carbon (DOC) yielded a relationship with *r*^2^ = 0.65, but DOC data were only available for eight samples, limiting the confidence in this finding. MCI baseline slope, an indicator of MSPM contamination, also showed a strong relationship with error.

**Figure 8.**
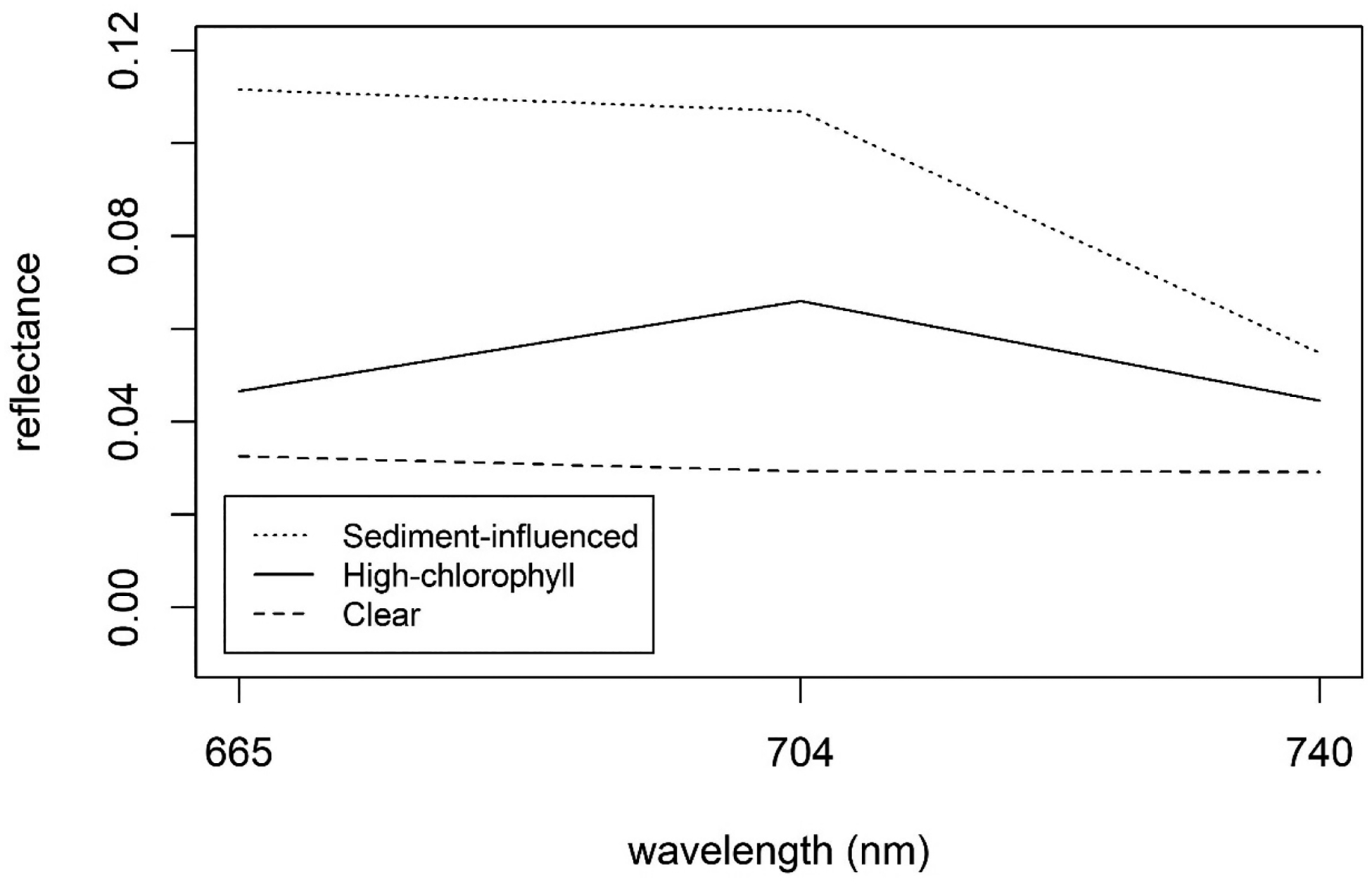
Example spectra of MSPM-influenced water (Lake Frederick, Oklahoma), high-chl *a* water (Dean Lake, Minnesota), and clear water (Lake Saint Croix, Wisconsin). The MSPM-influenced spectral line displays notably higher reflectance in band 4 than in band 6 (negative slope), while the clear and high-chl *a* waters display nearly uniform reflectance in bands 4 and 6.

**Figure 9.**
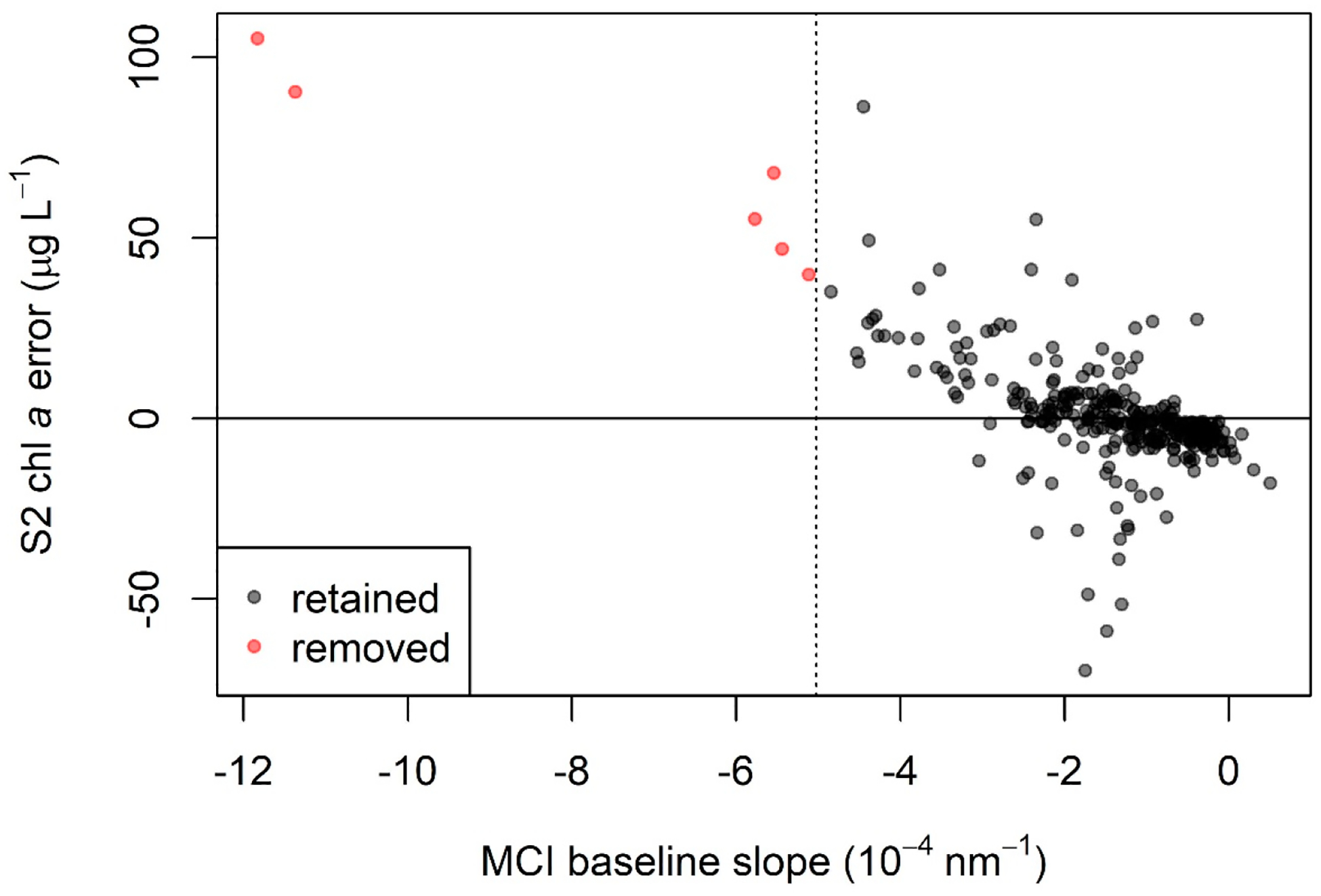
S2 chl *a* error (calculated as MCI chl *a* minus in situ chl *a*) as a function of MCI baseline slope for *ρ*_t_. More negative slope values represent a higher likelihood of MSPM contamination, leading to chl *a* overestimation, and are seen here with increasingly high error. The dotted line at MCI baseline slope = −1.5 × 10^−4^ nm^−1^ indicates the threshold below which samples were removed; points to the left of the dotted line were excluded from all analysis, including calibration and validation.

As discussed, MSPM is a major constituent that may interfere with MCI retrieval, causing elevated chl *a* retrievals [67]. The unique spectral characteristics of sediment-influenced waters are evident in the example spectra shown in Figure 8, with elevated reflectance in band 4 causing a negative baseline slope (see Figure 2). Ref. [67] found that MCI error was high when the baseline slope was more negative than −1.5 × 10^−4^ nm^−1^ for *R*_rs_. Since that study did not provide baseline slope thresholds for *ρ*_t_ and *ρ*_s_, we developed relationships between baseline slope for each of those processing levels and that of *R*_rs_ and used them to calculate baseline slope thresholds for *ρ*_t_ and to *ρ*_s_. These calculated thresholds were −5.0 × 10^−4^ nm^−1^ for *ρ*_t_ and −4.8 × 10^−4^ nm^−1^ for *ρ*_s_. The relationships between *R*_rs_ baseline slope and baseline slope for each of the other two processing levels were strong, both with *r*^2^ = 0.99, bolstering confidence that the threshold conversions were robust. Continuing the analysis using *ρ*_t_, we found that, indeed, S2 MCI heavily overestimated chl *a* for all six observations below the threshold, and error increased with more negative baseline slopes (Figure 9). Further inspection of Figure 9 shows that error was highly positive up to approximately −3 × 10^−4^ nm^−1^, suggesting that a more restrictive, less negative baseline slope threshold may further reduce error due to MSPM.

Another factor that may impact the MCI is proximity to land through adjacency effects and mixed pixel contamination. We used samples at least 30 m from the shore in an attempt to minimize these effects, similar to [94]. Still, remaining samples near the shore may have been impacted by land for several reasons. First, the static NHD lake shapefile used to establish lake boundaries does not always reflect the extent of water [95]. Due to fluctuations in water level and inaccuracies in the shapefile, land may be erroneously included. Second, water in land-adjacent pixels may be shallow enough that the sensor is capturing bottom reflectance, which may interfere with the MCI algorithm. Finally, land may cast stray light or shadows on nearby pixels [21,96], potentially impacting the spectra of such pixels and again interfering with the MCI. Ref. [97] discuss adjacency effects in S2 and present a correction that may be applied to mitigate them. The relationship between S2 chl *a* error and distance from shore was not strong in our data set, indicating that impacts on validation results were minimal. Thus, the adjacency effect correction presented in [97] was not applied here, but may be relevant in subsequent studies.

Ancillary data such as sun-sensor geometry information were investigated [29]. There was no discernible pattern in error relative to sensor angle, which ranged from 2 to 11 degrees from the zenith, nor in the solar angle, which ranged from 25 to 57 degrees from the zenith. Sun glint is another issue that may impact spectra and thus interfere with MCI implementation, particularly given the near-nadir view angle of S2 [98] and the relatively high solar-elevation angles associated with its overpass time [66]. Though some degree of glint may be present in the imagery utilized here, we did not remove potentially impacted images because they did not exhibit error that was systematically different than that of other sample locations. However, further investigation of the impact of glint on MCI is warranted, and methods to correct for glint in S2 imagery are available [98].

Clouds present a challenge for S2 applications. While many platforms are able to leverage thermal bands for cloud detection, S2 lacks such bands, presenting a limitation for developing effective masking [99]. Another factor that may obfuscate chl *a* retrievals is colored dissolved organic matter (CDOM). Ref. [100] showed that high CDOM may interfere with the MCI, generally resulting in underestimation of chl *a*. We did not investigate CDOM in our study, but, as mentioned, we did observe an inverse relationship between chl *a* error and DOC, albeit with a small sample size (*r*^2^ = 0.65; *n* = 8). For further discussion of the impact of CDOM on chl *a* detection in Case-2 waters, see [101]. Temporal difference between satellite overpass and in situ data collection presents another potential source of error, as phytoplankton may migrate laterally or within the water column between collection times. We attempted to minimize this error by constraining our analysis to in situ samples collected within 12 h on either side of satellite overpass. Though we observed no relationship between error and collection-time difference in our dataset, this remains a potential source of error. No appreciable difference was observed in S2 chl *a* error between the two satellites, Sentinel-2A and Sentinel-2B. ESA ensures calibration agreement within 5% between the two satellites and generally within 3% [102], a small quantity relative to the degree of error we observed in S2 chl *a* retrievals.

### 3.6. Application

Our results suggest that the S2 MCI approach is generally capable of identifying a range of chl *a* conditions across broad areas, enabling the application of the algorithm to lakes around the U.S. To demonstrate the use of the S2 MCI, we applied it to Jordan Lake, a eutrophic reservoir in central North Carolina (NC). Prior efforts have been made to use Landsat 8 to monitor chl *a* concentrations in Jordan Lake, with some success. Ref. [103] presents separate band ratio models for summer and fall with *r*^2^ of 0.73 and 0.48 and RMSE of 1.2 and 1.3 μg L^−1^, respectively. Ref. [104] employed a three-band approach to estimate chl *a* in Jordan Lake, reporting *r*^2^ of 0.66 and RMSE of 8.9 μg L^−1^.

**Figure 10.**
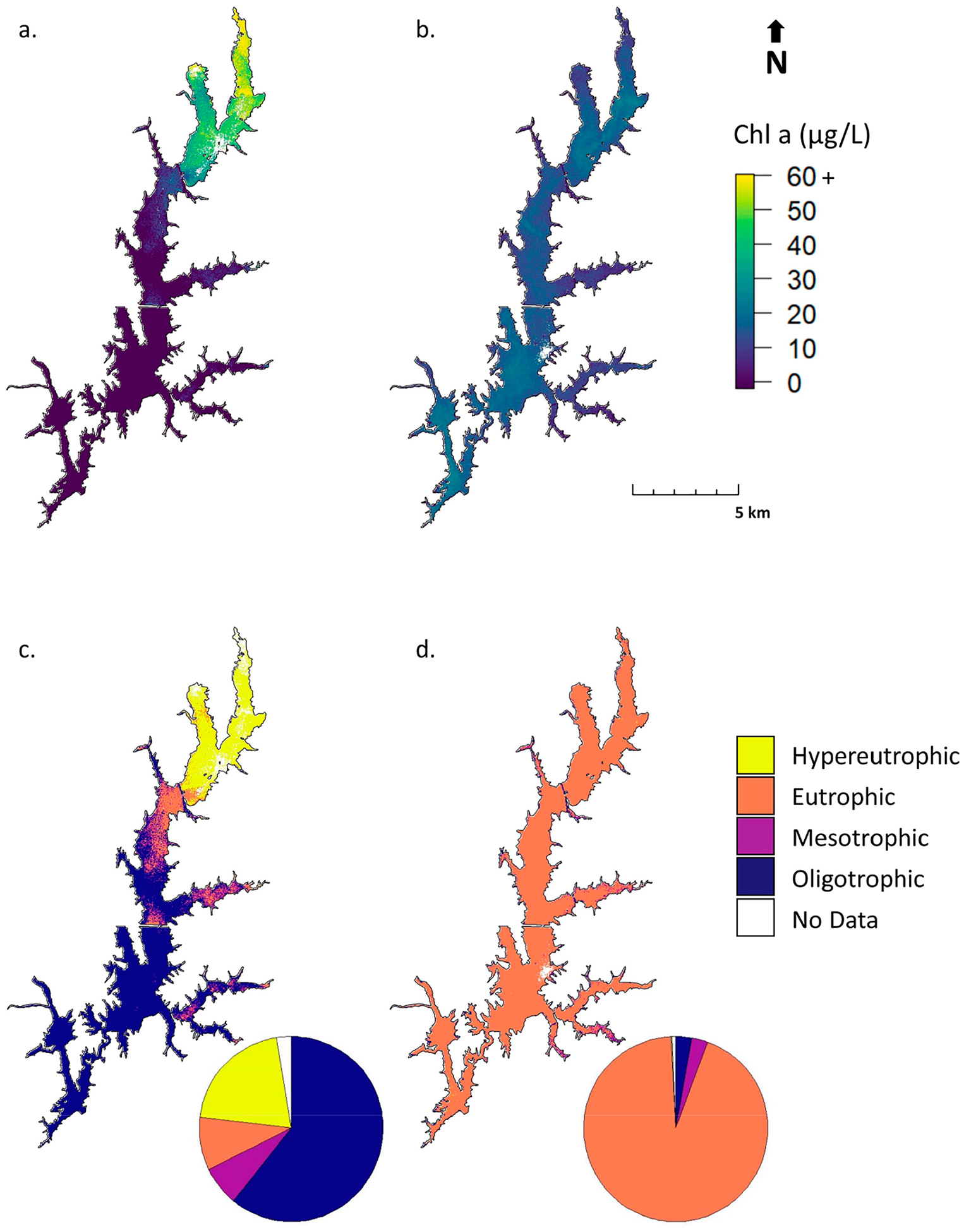
Example applications of the S2 MCI to display chl *a* (**a**,**b**) and trophic state (**c**,**d**) in Jordan Lake, NC on 14 May (**a**,**c**) and 1 October (**b**,**d**), 2018. Pie charts indicate the relative proportions of each trophic state. Data with high MSPM contamination have been removed and are shown as No Data. See Table 2 for chl *a* ranges equating to each trophic state.

Figure 10 illustrates an application of the S2 MCI to generate chl *a* estimates (Figure 10a,b) and visualize the trophic state (Figure 10c,d) in Jordan Lake using imagery from May 14 and October 1, 2018. The trophic state is often used to qualitatively assess nutrient-loading and algae-bloom susceptibility and was here determined based on the thresholds in Table 2. Pie charts show the relative proportions of each trophic state class. Imagery from May 14 shows broad spatial variation in chl *a* values, with higher concentrations near some inflows. On October 1, chl *a* is more spatially consistent across the lake, with nearly complete eutrophic status. These figures provide an example of how S2 imagery can be used to investigate variations in chl *a* and trophic status across time and space.

The 2018 NC Department of Environmental Quality (NCDEQ) report contains annual summary results for monthly chl *a* sampling at nine sites in Jordan Lake [84]. Comparing chl *a* values from S2 imagery to this summary provides a sense of whether retrieved satellite values are realistic, though this approach is anecdotal since the data from specific sampling dates are unavailable. Chl *a* values in Figure 10a,b are generally within the ranges shown in the report, with some exceptions. NCDEQ reported chl *a* values for three sites in the northern section of the lake ranging from 18 to 110 μg L^−1^, with annual mean chl *a* concentrations of 46 to 51 μg L^−1^. S2 chl *a* near those sites from May 14 ranges from 10 to 39 μg L^−1^—below the mean but generally within the range reported—while S2 chl *a* values from October 1 range from 10 to 17 μg L^−1^—slightly below the range. At three sites in the middle portion of the lake, NCDEQ reported concentrations ranging from 13 to 51 μg L^−1^, with annual means between 29 and 37 μg L^−1^. S2 chl *a* estimates from May 14 ranged from below detection to 15 μg L^−1^, and values from October 1 ranged from 12 to 19 μg L^−1^. At three sites in the Haw River arm, the southwestern region of Jordan Lake observable as a spur stretching northwest, NCDEQ reported chl *a* ranging from 5 to 100 μg L^−1^, with annual means of 19 to 31 μg L^−1^. S2 estimates from May 14 were below the detection limit, and from October 1, 12 to 21 μg L^−1^—just below mean values and well within the overall range. The comparison suggests that some underestimation may be occurring, but this is inconclusive, given the limited frequency of the NCDEQ sampling and the fact that the specific value of the satellite detection limit is at this time unquantified. Overall, S2 chl *a* values reflect spatial patterns across Jordan Lake exhibited by the NCDEQ in situ data.

Though the MCI and NDCI have been available to water-quality managers for years, broad-scale parameterization of these algorithms as presented in this study enables the development of a national chl *a* estimation product. The Cyanobacteria Assessment Network (CyAN) has done so on an operational basis for cyanobacteria abundance using S3 OLCI, providing a publicly available product utilized by state agencies and various other lake management stakeholders [95]. Given its spatial resolution of 300 m and a one-pixel buffer adjacent to land, S3 provides data for less than 0.7% of the 275,897 waterbodies across CONUS in the NHDPlus database. An approach similar to CyAN could be developed for S2, which can provide data for 98.8% of those waterbodies as well as many rivers and estuaries. Such a product would provide enormous value on its own, but could also be harmonized with S3 products—whether CyAN or a complementary chl *a* product such as that presented in [17]—which would greatly expand the temporal frequency of observations. Additionally, planning for the Landsat Next missions indicates that the platform will offer similar spectral bands and resolution to those of S2, providing opportunities for more seamless harmonization and increased temporal frequency. Adding a 685 nm band to S2 and/or Landsat Next would allow more seamless standardization with S3 and MERIS products, enabling the use of algorithms such as the Cyanobacteria Index, Fluorescence Line Height, and Maximum Peak Height algorithms.

## 4. Conclusions

This study demonstrated the feasibility of estimating chl *a* concentrations in freshwater lakes and reservoirs on a broad scale by applying the MCI or NDCI to S2 data. Both algorithms have the ability to estimate chl *a* concentration over a wide range of conditions, enabling transformation of those estimates into trophic state. Generally, MCI outperformed NDCI by a small margin. This work parameterized, assessed error of, and demonstrated the application of these algorithms, previously only validated at a select-few individual lakes, on a broad spatial (103 lakes) and temporal (4 years) scale. This study contributes toward algorithm maturity, transitioning from accuracy assessment using a small number of measurements to employing a larger, more spatiotemporally expansive dataset. This provides an opportunity for stakeholders who request higher resolution chl *a* satellite data, paving the way for further testing and the adoption of S2 use in small lakes—areas where coarser-resolution satellite platforms are often unable to provide data. While it was previously suggested that parameterization is required for each optical water type [105] or local system [106], we demonstrate that the use of a single set of parameters is viable for trophic state determination across a range of systems. Future integration with optical water-type classification will provide advances in algorithm performance, but the necessary radiometric and in situ optical measures are sparse. Most management groups do not have the resources to parameterize by system or the technical resources to quantify optical water types, let alone implement the necessarily complex processing demands [107]. Facilitating the use of off-the-shelf approaches, such as those demonstrated here, may reduce development and implementation requirements. There are still challenges to effective remote sensing of inland waters, such as limited availability of in situ data, issues with atmospheric correction, and operational capacity [108]. This study emphasized the complexity of addressing these challenges and demonstrated that doing so could create great capacity for broad-scale remote sensing of inland waters with S2.

## Figures and Tables

**Table 1.** Comparison of S2 bands and MERIS bands used in MCI calculation.

Band	MERIS			S2		
	Band Number	Band Center (nm)	Band Width (nm)	Band Number	Band Center (nm)	Band Width (nm)
left baseline (a)	8	681	7.5	4	665	31
peak (b)	9	709	10	5	705	15/16 *
right baseline (c)	10	753	7.5	6	741/739 *	15

* values for S2A and S2B, respectively.

**Table 2.** Chl *a* ranges for each trophic state defined in the National Lakes Assessment.

Trophic State	Chl a Concentration (μg L^−1^)
Oligotrophic	≤2
Mesotrophic	>2 and ≤7
Eutrophic	>7 and ≤30
Hypereutrophic	>30

**Table 3.** Summaries of in situ chl *a*, MCI, and NDCI values in calibration (Cal.) and validation (Val.) datasets. Validation datasets were slightly different for MCI and NDCI since each algorithm resulted in different numbers of negative chl *a* observations, which were removed.

Set	*n*	# Lakes	# States	In Situ Chl *a* (μg L^−1^)				MCI, *ρ*_t_				NDCI, *ρ*_t_			
				Min	Med	Mean	Max	Min	Med	Mean	Max	Min	Med	Mea n	Max
All	294	103	28	0.1	6.4	13.8	132.4	−0.0044	0.0005	0.0022	0.0278	−0.141	−0.058	−0.050	0.148
Cal.	235	93	26	0.1	6.0	13.0	105.0	−0.0044	0.0003	0.0019	0.0243	−0.140	−0.057	−0.051	0.142
Val. MCI	44	19	10	0.0	12.0	21.4	132.4	−0.0017	0.0023	0.0052	0.0278	-	-	-	-
Val. NDCI	49	30	12	0.42	8.1	18.8	132.4	-	-	-	-	−0.084	−0.054	−0.031	0.148

**Table 4.** Calibration coefficients obtained during model calibration, with 95% confidence intervals (lower and upper—LCI and UCI, respectively), and validation error metrics between S2 chl *a* and in situ chl *a* for each of the three processing levels and both investigated algorithms. Calibration *n* varies due to different numbers of observations removed based on MSPM contamination; validation *n* varies for the same reason, and also because each relationship resulted in a different number of negative chl *a* values, which were removed prior to error analysis. *R*_rs_ NDCI contained extreme values that generated high uncertainty and are italicized to advise use of caution in interpretation.

Algorithm	Proc. Level			Calibration						Validation			
		*β*_1_	*β*_1_ LCI	*β*_1_ UCI	*β*_0_	*β*_0_ LCI	*β*_0_ UCI	*r* ^2^	*n*	MAE_mult_	Bias_mult_	MAPE	*n*
MCI	*ρ*_t_	3586	2802	42354	6.27	5.26	7.87	0.50	235	2.08	1.15	100.9	44
	*ρ*_s_	3441	2704	4057	6.17	5.10	7.85	0.49	235	2.22	1.05	100.1	46
	*R*_rs_	9043	6431	10,878	5.99	4.92	7.34	0.47	236	2.47	1.17	307.3	49
NDCI	*ρ*_t_	391	294	476	33.1	27.0	38.9	0.46	235	2.41	1.52	220.1	49
	*ρ*_s_	240	151	288	18.5	14.8	21.4	0.31	235	3.12	1.51	323.4	48
	*R*_rs_	62	−*92.5*	*4336*	*10.2*	−*3230*	*18.5*	*0.02*	*236*	*2.68*	*1.53*	*318.4*	*48*

**Table 5.** Confusion matrix showing accuracy of S2 chl *a* trophic state for *ρ*_t_ MCI. Accurately identified quantities are bolded. Overall classification accuracy was 66%. Non-bold values in the same column indicate erroneously excluded observations, and non-bold values in the same row indicate erroneously included observations.

		Reference (In Situ Chl *a*)			
		Oligotrophic	Mesotrophic	Eutrophic	Hypereutrophic
Prediction (S2 chl *a*)	oligotrophic	**2**	1	0	0
	mesotrophic	0	**4**	2	0
	eutrophic	1	5	**15**	2
	hypereutrophic	0	0	4	**8**

**Table 6.** Error metrics for *ρ*_*t*_ MCI by trophic state (defined by in situ values). MAE_add_ and bias_add_ refer to traditional additive metrics, while MAE_mult_ and bias_mult_ refer to multiplicative metrics used throughout the study.

Trophic State	MAE_add_ (μg L^−1^)	Bias_add_ (μg L^−1^)	MAE_mult_	Bias_mult_	MAPE	n
Oligotrophic	2.6	1.5	7.35	0.46	231%	3
Mesotrophic	6.2	4.8	2.37	1.48	126%	10
Eutrophic	13.0	8.8	1.88	1.33	99%	21
Hypereutrophic	23.9	−7.4	1.54	0.87	40%	10

## Data Availability

Data used to generate the results herein are available at the following DOI: https://doi.org/10.23719/1529420 (accessed on 1 May 2024).

## Appendix A

**Table A1.** Confusion matrix showing accuracy of S2 chl *a* trophic state for *ρ*_t_ NDCI. Accurately identified quantities are bolded. Overall classification accuracy was 47%. Non-bold values in the same column indicate erroneously excluded observations, and non-bold values in the same row indicate erroneously included observations.

		Reference (In Situ Chl *a*)			
		Oligotrophic	Mesotrophic	Eutrophic	Hypereutrophic
Prediction (S2 chl *a*)	**oligotrophic**	**1**	1	0	0
	**mesotrophic**	2	**2**	3	0
	**eutrophic**	5	10	**12**	2
	**hypereutrophic**	0	0	3	**8**

**Table A2.** Error metrics for *ρ*_*t*_ NDCI by trophic state (defined by in situ values). MAE_add_ and bias_add_ refer to traditional additive metrics, while MAE_mult_ and bias_mult_ refer to multiplicative metrics used throughout the study.

Trophic State	MAE_add_ (μg L^−1^)	bias_add_ (μg L^−1^)	MAE_mult_	bias_mult_	MAPE	n
Oligotrophic	7.4	7.3	6.73	6.33	864%	8
Mesotrophic	6.9	6.6	2.78	1.94	175%	13
Eutrophic	9.7	3.2	1.80	0.95	71%	18
Hypereutrophic	22.0	−10.3	1.49	0.82	31%	10
